# Endophytic non-pathogenic *Fusarium oxysporum* reorganizes the cell wall in flax seedlings

**DOI:** 10.3389/fpls.2024.1352105

**Published:** 2024-03-25

**Authors:** Wioleta Wojtasik, Lucyna Dymińska, Jerzy Hanuza, Marta Burgberger, Aleksandra Boba, Jan Szopa, Anna Kulma, Justyna Mierziak

**Affiliations:** ^1^ Department of Genetic Biochemistry, Faculty of Biotechnology, Wroclaw University, Wroclaw, Poland; ^2^ Department of Bioorganic Chemistry, Wrocław University of Economics and Business, Wrocław, Poland; ^3^ Institute of Low Temperature and Structure Research, Polish Academy of Sciences, Wrocław, Poland

**Keywords:** flax, seedlings, endophyte, Fusarium oxysporum, cell wall

## Abstract

**Introduction:**

Flax (*Linum usitatissimum*) is a crop producing valuable products like seeds and fiber. However, its cultivation faces challenges from environmental stress factors and significant yield losses due to fungal infections. The major threat is *Fusarium oxysporum* f.sp *lini*, causing fusarium wilt of flax. Interestingly, within the *Fusarium family*, there are non-pathogenic strains known as biocontrols, which protect plants from infections caused by pathogenic strains. When exposed to a non-pathogenic strain, flax exhibits defense responses similar to those seen during pathogenic infections. This sensitization process activates immune reactions, preparing the plant to better combat potential pathogenic strains. The plant cell wall is crucial for defending against pathogens. It serves as the primary barrier, blocking pathogen entry into plant cells

**Methods:**

The aim of the study was to investigate the effects of treating flax with a non-pathogenic *Fusarium oxysporum* strain, focusing on cell wall remodeling. The infection’s progress was monitored by determining the fungal DNA content and microscopic observation. The plant defense response was confirmed by an increase in the level of Pathogenesis-Related (PR) genes transcripts. The reorganization of flax cell wall during non-pathogenic *Fusarium oxysporum* strain infection was examined using Infrared spectroscopy (IR), determination of cell wall polymer content, and analysis of mRNA level of genes involved in their metabolism.

**Results and discussion:**

IR analysis revealed reduced cellulose content in flax seedlings after treatment with Fo47 and that the cellulose chains were shorter and more loosely bound. Hemicellulose content was also reduced but only after 12h and 36h. The total pectin content remained unchanged, while the relative share of simple sugars and uronic acids in the pectin fractions changed over time. In addition, a dynamic change in the level of methylesterification of carboxyl groups of pectin was observed in flax seedlings treated with Fo47 compared to untreated seedlings. The increase in lignin content was observed only 48 hours after the treatment with non-pathogenic *Fusarium oxysporum*. Analysis of mRNA levels of cell wall polymer metabolism genes showed significant changes over time in all analyzed genes. In conclusion, the research suggests that the rearrangement of the cell wall is likely one of the mechanisms behind flax sensitization by the non-pathogenic *Fusarium oxysporum* strain. Understanding these processes could help in developing strategies to enhance flax’s resistance to fusarium wilt and improve its overall yield and quality.

## 1 Introduction

Flax (*Linum usitatissimum*) is a versatile crop providing valuable fiber and oil. Traditionally flax fibres was used for textiles and chemical industry applications, it now finds innovative use in dressings for hard-to-heal wounds, biodegradable packaging, tissue engineering scaffolds, and surgical threads (Skórkowska-Telichowska et al., 2010; Czemplik et al., 2011; Kulma et al., 2015). Flax seeds offer rich nutrition with polyunsaturated fatty acids, phytosterols, vitamins, lignans, and squalene, combating diseases like atherosclerosis and hypertension (Shim et al., 2014; Kajla et al., 2015; Zuk et al., 2015). Additionally, byproducts of flax processing like seedcakes and shives possess antioxidant, antibacterial, antifungal, and anticancer properties (Zuk et al., 2014; Czemplik et al., 2017).

The occurrence of yield losses in flax crops can be attributed to adverse climatic conditions, although the most significant impact is exerted by fungi belonging to the genus *Fusarium* (Heller et al., 2006). Among these, *Fusarium oxysporum* f.sp *lini* stands out as the most perilous pathogen, provoking fusarium wilt of flax. Fusarioses, besides diminishing flax yield, also manifest in the depreciation of fiber and seed quality, along with their derived products (Olivain and Alabouvette, 1997).

There exist strains of *Fusarium oxysporum* that exhibit an intriguing behavior of penetrating and colonizing the root structure without inducing any vascular system attacks or infections. These strains are commonly referred to as non-pathogenic or endophytic. Among the most renowned strains in this category are Fo47 and CS-20. A marked distinction between these endophytic strains and pathogenic counterparts lies in their effector gene composition, host colonization mechanisms, localization within the plant, and their ability to induce host responses. While endophytic strains elicit host immune reactions, such as localized cell death in the root cortex or activation of immune signaling pathways, these mechanisms are largely suppressed by pathogenic strains (De Lamo and Takken, 2020).

During the initial stages of colonization, both pathogenic and non-pathogenic strains of *F. oxysporum* exhibit striking similarities in their behavior. In either case, contact with the root triggers hyphal proliferation and branching, eventually leading to the development of specialized hyphal swellings that facilitate further root penetration. The entry of spores into the root occurs through minute micro-damages, particularly at vulnerable points like the emergence sites in lateral roots, or through direct penetration of the root tip. It should be noted that these mechanisms can vary depending on the specific *F. oxysporum* species and the type of infected plant. For the pathogenic strain of *F. oxysporum*, studies conducted on flax plants have revealed that its root penetration is limited to the apex, targeting undifferentiated tissues (Turlier et al., 1994). The hyphae of the pathogen reach the vascular stele through the apoplast of the root cortex. Moreover, during intracellular proliferation, local cell death has been observed, although this phenomenon tends to occur more frequently in non-pathogenic strains (Gordon, 2017; De Lamo and Takken, 2020).

One of the differences between pathogenic and non-pathogenic strains is the amount of fungal biomass formed in the root. In the early stages, endophytic strains of *F. oxysporum* are less efficient colonizers than pathogens. Two weeks after tomato inoculation, the biomass of the pathogenic strain was 10 times higher (Lanubile et al., 2015). Another significant difference is the colonization pattern. While the colonization of non-pathogenic Fo47 is limited to the surface of the root and the outer layers of cortical cells, the pathogen intensively attacks deeper root tissues, eventually reaching the vessels (Olivain and Alabouvette, 1997; De Lamo and Takken, 2020).

Non-pathogenic strains frequently possess the capability to impede infections instigated by pathogenic strains. They achieve this through diverse mechanisms, such as competing for nutrients in the soil, consequently influencing the germination rate of the pathogen’s chlamydospores. Additionally, non-pathogenic strains engage in competition for infection sites both within and on the root structure. Furthermore, these strains may induce plant resistance, a phenomenon known as endophyte-mediated resistance (EMR) (Fravel et al., 2003; Ajilogba and Babalola, 2013; Constantin et al., 2019). Consequently, the presence of the non-pathogenic strain induces a sensitization process due to its activation of the plant’s immune response. The plant becomes better equipped to combat potential encounters with pathogenic strains, not only through competitive exclusion from shared habitats but also by possessing a higher abundance of enzymes like chitinase and β-1,3-glucanases, which directly counteract fungal infections by decomposing the pathogen cell wall and swiftly arresting the spread of infection. As a result of this sensitization mechanism, the plant achieves improved adaptation to environmental conditions, leading to enhanced overall condition and resilience (Olivain and Alabouvette, 1997; Fravel et al., 2003; Wojtasik et al., 2019).

The plant cell wall plays a key role in defending against an invading pathogen. It is the plant’s first line of defense and acts as a physical barrier that hinders or inhibits pathogen penetration into plant cells. The cell wall is composed of polysaccharide polymers (cellulose, hemicellulose and pectin) and non-polysaccharide polymers (lignin), as well as structural and enzymatic proteins (Keegstra, 2010; Pavitra et al., 2019). When infected, pathogenic microorganisms secrete cell wall digesting enzymes (CWDEs), such as pectinases, cellulases and hemicellulases. Pectin methylesterases are responsible for the deesterification of pectin, which leads to changes in the porosity and structure of the cell wall, enabling polygalacturonases and pectin lyases to depolymerize pectin, and then cellulases and hemicellulases to degrade cellulose and hemicellulose (Kang and Buchenauer, 2000; 2002;Lionetti et al., 2012; Kubicek et al., 2014; Santos et al., 2014). The degradation of polysaccharides by pathogens yields nutrients, notably glucose. Pathogen-induced oligosaccharides (OGs) and pectin fragments serve as elicitors, activating plant defense mechanisms. This leads to the production of pathogenesis-related proteins, including β-1,3-glucanases and chitinases, which degrade components of the fungal cell wall, enhancing plant resistance against subsequent infections (Petruzzelli et al., 1999; Cheong et al., 2000; Li et al., 2001; Wu and Bradford, 2003; Berlin et al., 2007; Ferrari et al., 2008; Lionetti et al., 2010; Balasubramanian et al., 2012; Ferrari et al., 2013). During infection, some pathogens manipulate the activity of host genes involved in pectin degradation, facilitating easier access to further pectin breakdown (Hok et al., 2010; Lionetti et al., 2012).

Pathogen infection can also trigger increased lignin biosynthesis through the phenylpropanoid pathway, reinforcing the cell wall and providing enhanced protection against pathogen attacks. The defense reactions are mediated by a signal cascade induced by reactive oxygen species (ROS), which play roles as signal molecules and electron acceptors during lignification (Bhuiyan et al., 2009; Collinge, 2009). Hemicelluloses, particularly xylans, contribute to cell wall reinforcement, and their acetylation modification affects plant resistance to pathogen infection (Escudero et al., 2017; Gao et al., 2017).

This study aimed to investigate the involvement of cell wall polymers (cellulose, hemicellulose, pectin, and lignin) in flax in response to treatment with a non-pathogenic strain of *F. oxysporum*. The analysis focused on observing alterations in the cell wall structure, considering the content of individual polymers and the expression patterns of genes involved in their metabolism. Prior to this research, the existence of non-pathogenic *F. oxysporum* in flax tissues was confirmed through microscopic observations and amount of fungal DNA. Additionally, the defense response of flax plants was validated by analyzing the transcript levels of PR genes, specifically chitinase and β-1,3-glucanases.

## 2 Materials and methods

### 2.1 Biological material and growth conditions

Flax seeds (*Linum usitatissimum* L., cv. Nike) were sourced from the Flax and Hemp Collection at the Institute of Natural Fibres in Poland. The seeds were sterilized using a 70% ethanol for 5 minutes and 50% PPM (Plant Cell Technology, UK) solution for 10 minutes and then placed onto Petri dishes containing MS medium supplemented with 0.8% agar and 1% sucrose. The Petri dishes were used for seedling growth, which took place for 12 days in a controlled environment within a plant growth chamber. The growth chamber maintained a 16-hour light period at 21°C, followed by an 8-hour darkness period at 16°C. These seedlings were later utilized for subsequent experiments.

For the experiments, a non-pathogenic strain of *Fusarium oxysporum* (ATCC Number: MYA-1198) was acquired from ATCC (USA). The *Fusarium fungus* was cultured on Petri dishes with PDA medium at a temperature of 28°C for 14 days. After flooding the plate with 1 ml of sterile water, a mixture of macro- and microconidia spores was harvested. The spores were then counted using a hemocytometer and diluted to a concentration of 10^6^ spores/ml for the inoculation process.

### 2.2 Flax seedlings treatment with non-pathogenic *Fusarium oxysporum* strain

To initiate the experiment, 200 μl of *F. oxysporum* inoculum at a concentration of 10^6^ spores/ml was evenly spread onto Petri dishes containing potato dextrose agar (PDA) medium. These dishes were then cultivated at 28°C for 4 days. Meanwhile, 12-day-old flax seedlings, along with the medium, were carefully transferred to separate Petri dishes with PDA medium, one containing the non-pathogenic strain of *F. oxysporum*, and the other serving as a control without the fungus. Both the control and infected samples were cultivated in the same chamber under controlled conditions.

At specific time intervals (3, 6, 12, 24, 36, and 48 hours), the treated and non-treated (control) seedlings were collected separately, rapidly frozen in liquid nitrogen, and stored in a deep freezer at -80°C for further analysis. The entire experiment was conducted in three independent biological repetitions to ensure reliability and reproducibility of the results.

### 2.3 Microscopic observation of the progress of fungal infection

Whole plants were collected and treated with 0.15% TCA in ethanol:chloroform mixture (4:1, v/v) for 48 h. The roots were subjected to a series of washes: sterile water (1x), 50% ethanol (2x), 50mM NaOH (2x), sterile water (1x) for 15 minutes each, then in 0.1M Tris-HCl buffer, pH=8.5 for 30 minutes. After a series of washes, the tissue was stained. Roots were submerged in safranin solution (0.2% w/v safranin in 10% v/v ethanol) for 3 min and washed three times in water after 10 min. The test material was cut into sections with a razor blade and mounted on the slides. Subsequently, sections on the slides were stained for 10 min with solophenyl flavine 7GFE (0.1% w/v in 0.1 M Tris/HCl, pH 8.5), and washed again with water (3 times). Samples were observed under an epifluorescence microscope (ZEISS AXIO Scope A.1.) using a UV filter, (360–370 nm excitation wavelength), at 40x magnification. Images were documented using the black and white camera AxioCam ICm1 and ZEISS ZEN 2.6. lite software. Colors were added digitally after the photo was taken to enhance visualization.

### 2.4 Identification of fungal DNA in plant material

Genomic DNA (plant and fungi) was isolated using DNeasy Plant Mini Kit (QIAGEN) following the manufacturer’s protocol. The DNA integrity was examined by gel electrophoresis on 1.0% (w/v) agarose and the DNA amount was determined with the spectrophotometric method.

The DNA (100 ng/µl) was used as a template for the PCR reaction on a 152 bp fragment of Fo47 genome (primers: Fwd: CCAAGGCAGAAGTCGATGTA and Rev: AGGTCGTTGGTGAGAAAG) and for the q-PCR reaction on a 150 bp fragment of fungal murein transglycosylase gene (primers: Fwd: TCTCAACGGTGTCGAGTCTAA and Rev: CACCCTGGTTGCAGATAAT). Actin gene was used for reference.

The first PCR reaction using primers for a fragment of Fo47 genome and Color Taq PCR Master Mix (EURX, Poland) was performed in the BIO RAD - PTC-200 DNA Engine Peltier Thermal Cycler. Program: 95°C, 3:00min; 95°C, 0:30min; 59°C, 0:30; 72°C, 0:05min; 72°C, 5:00 min. PCR products were subjected to electrophoretic separation in a 2% agarose gel and at voltages adjusted to the size of the separated DNA, in TBE buffer (90mM Tris, 90mM boric acid, 20mM EDTA). The separated DNA was visualized under UV light.

The second PCR reactions were performed in the Applied Biosystems StepOnePlus Real-Time PCR system using the qPCR Master Mix (2x) reagent kit (EURx, Poland). The reaction conditions were: 95°C for 15 min (holding stage); and 95°C for 10 s, 57°C for 20 s, 72°C for 30 s, 37 cycles (cycling stage). The conditions of the melting curve stage were: 95°C for 15 s, 60°C for 1 min, 95°C for 30 s, and ramp rate: 1.5%.

### 2.5 The mRNA levels analysis

The real-time PCR technique was employed to determine the mRNA levels of the genes under investigation. Total RNA was extracted using the TRIzol method as per the manufacturer’s protocol, and the RNA’s integrity was assessed by gel electrophoresis on a 1% (w/v) agarose gel containing 15% (v/v) formaldehyde. Any remaining DNA was eliminated through DNase I treatment. Subsequently, the RNA was used as a template for cDNA synthesis, employing the High Capacity cDNA Reverse Transcription Kit (Thermo Fisher Scientific, U.S).

For the q-PCR reactions, a qPCR Master Mix (2x) reagent kit (EURx, Poland) was utilized on an Applied Biosystems StepOnePlus Real-Time PCR System. The reaction conditions were designed following the instructions provided by the kit manufacturer. The reaction conditions were: 95°C for 15 min (holding stage); and 95°C for 10 s, 57°C for 20 s, 72°C for 30 s, 37 cycles (cycling stage). The conditions of the melting curve stage were: 95°C for 15 s, 60°C for 1 min, 95°C for 30 s, and ramp rate: 1.5%. The primers were designed using LightCycler Probe Design Software 2, and their specificity at the designated temperature was confirmed by analyzing the PCR products using the melting curve method (**Supplementary Table 1**). Each reaction was conducted in three replicates. To normalize the data, the actin gene was used as a reference gene. Changes in mRNA levels were presented as x-fold relative quantities (RQ) standardized for actin (the reference gene) in comparison to the control, non-treated plants.

### 2.6 Content and structure of cell wall polymers

Cellulose content was determined with the anthrone method described by Ververis (Ververis et al., 2004). Isolation and fractionation of cell wall polysaccharides were performed with modified methods described by Manganaris and Vicente (Manganaris et al., 2008). Total pectin content was assayed as the sum of simple sugars from all fractions of cell wall pectin (WSF, CSF and NSF). Similarly, hemicellulose content was assayed by summing the simple sugars from fractions K1SF and K4SF. The content of total simple sugars was assayed with the phenol-sulfuric acid method after previous hydrolysis of polysaccharides with sulfuric acid (Ahmed and Labavitch, 1978). 600 μl of concentrated sulfuric acid was added to 300 μl of the supernatant, mixed and supplemented with 50 μl of 5% phenol aqueous solution. The samples ware incubated for 20 min at 50°C, and then, after cooling down, the simple sugar content was assayed spectrophotometrically at λ=480 nm. Glucose was used for standard curve preparation. Uronic acid content was assayed with the biphenyl method (Blumenkrantz and Asboe-Hansen, 1973) after polysaccharide hydrolysis with sulfuric acid (Ahmed and Labavitch, 1978). Glucuronic acid was used for standard curve preparation. The total lignin content was determined with the acetyl bromide method described by Iiyama and Wallis (Iiyama and Wallis, 1990). All methods used to determine the content of cell wall polymers are fully described in publication (Wojtasik et al., 2016).

Infra-red spectroscopy - FTIR/ATR spectra were recorded in the 4000–400 cm^-1^ range using a Nicolet 6700 spectrometer (Thermo Fisher Scientific, Waltham, USA) equipped with a portable ATR set. The resolution of these measurements was 2 cm^-1^. All FTIR spectra were processed in the same way before the statistical analysis. In the first step, the spectra were compared and analyzed by the commercial computer software (OriginPro). This analysis included background subtraction, using an internal standard, and the deconvolution of the experimental bands into Lorentz components. The intensity of the band at 2920 cm^-1^ was an internal standard. This band corresponds to the ν_as_(CH_3_) vibration. The Principal Component Analysis (PCA) was performed using the OriginPro 2024 (OriginLab Corp., Northampton, MA, USA).

X-ray diffraction - The XRD patterns of powder samples were recorded within the 2*Q* range 5-50° on an EMPYREAN II diffractometer (PANalytical) with Cu*Kα*_1,2_ radiation (*Λ*_aver_=1.5418 Å, the scanning step 0.013°). The High-Score Plus ver. 4.0 software was applied to analyze recorded powder patterns. The crystallinity of studied samples was determined from diffractograms based on the relation between the global peaks area and the reduced peaks area assigned to the crystalline part of the sample and expressed in percentage. Calculation of the CI followed the equation CI (%) = A_II_- A_I_/A_II_ × 100, where A_II_ is the integral intensity at 2θ=22°, and A_I_ is the integral intensity at 2θ=17.5° for samples with maximum intensity at 2θ=17.5°. But for samples with maximum intensity at 20° A_II_ is the integral intensity at 2θ=25°, and A_I_ is the integral intensity at 2θ=20°.

## 3 Results

### 3.1 Progression of infection with non-pathogenic *F. oxysporum* in flax seedlings

In order to confirm the infection of flax seedlings by the non-pathogenic *Fusarium oxysporum* (Fo47) strain, microscopic observations were carried out (**Supplementary Figure 1**). After 3 hours from the treatment, the presence of the fungus was not detected in the tested plant material. Single Fo47 hyphae were observed after 6 hours. 12 hours after treatment very numerous fungal hyphae were visible. In the following hours, a numerous network of fungal hyphae was also observed. No non-pathogenic strain of *F. oxysporum* was detected in the control plants.

**Figure 1:**
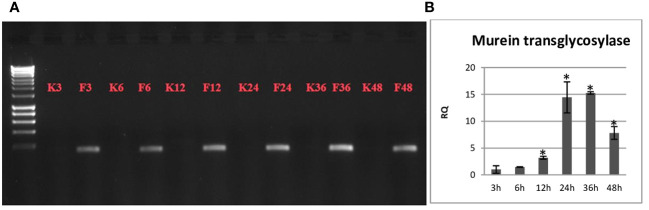
**(A)** Electrophoresis of the fragment of the Fo47 genome sequence obtained after the PCR reaction. **(B)** The level of the murein transglycosidase gene of the fungus in Nike flax seedlings treated with a non-pathogenic strain of *Fusarium oxysporum* over time (3 h, 6 h, 12 h, 24 h, 36 h and 48 h) are presented as the relative amount (RQ) in relation to the flax actin gene. Fo47 3h = 1. Results were obtained by real-time PCR on a DNA template and are presented as mean ± SD (n=3). The Student’s t-test was used to determine the statistical significance of the obtained results (* -P < 0.05).

The presence of the non-pathogenic *F. oxysporum* strain in flax seedlings was confirmed by PCR by amplifying a fragment of the Fo47 genome sequence and by q-PCR by amplifying the murein transglycosidase gene of the fungus (**Figure 1**). The non-pathogenic strain was detected in the flax seedlings at the 3-hour time point after treatment and exhibited a gradual increase in abundance over the duration of the observation period. This temporal variation in the population of the non-pathogenic strain was validated through q-PCR analysis, which revealed a time-dependent escalation, peaking at 24 and 36 hours, where its abundance was approximately 15 times higher compared to the 3-hour time point.

### 3.2 Transcript levels of PR genes in flax seedlings treatment with a non-pathogenic strain of *Fusarium oxysporum*

**Figure 2:**
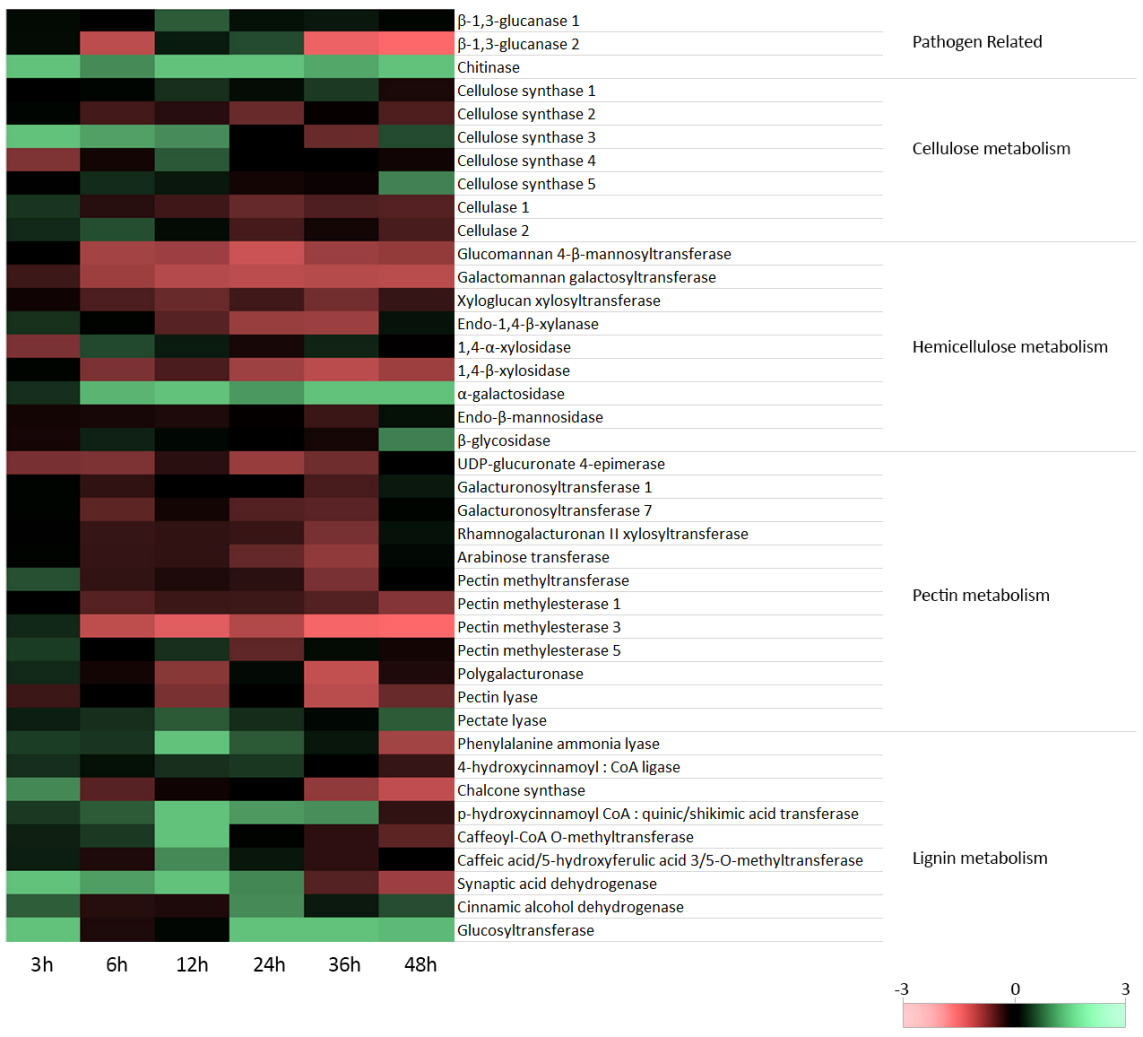
Expression of cell wall polymer metabolism genes in flax seedlings in response to non-pathogenic strain of *Fusarium oxysporum*. Changes in the levels of transcript of β-1,3-glucanase 1 and 2 and chitinase genes, cellulose synthase gene isoforms 1-5, genes of cellulose degradation (cellulase 1 and 2), hemicellulose synthesis (glucomannan 4-β-mannosyltransferase, galactomannan galactosyltransferase and xyloglucan xylosyltransferase) and hemicellulose degradation (endo-1,4-β-xylanase, 1,4-α-xylosidase, 1,4-β-xylosidase, α-galactosidase, endo-β-mannosidase and β-glycosidase), pectin synthesis (UDP-D-glucuronate 4-epimerase, galacturonate transferase 1, galacturonate transferase 7, xylose:rhamnogalacturonate transferase II, arabinose transferase, pectin methyltransferase) and pectin degradation (pectin methylesterase 1, pectin methylesterase 3, pectin methylesterase 5, polygalacturonase, pectin lyase I and pectin lyase II), and lignin metabolism (phenylalanine ammonia lyase, 4-hydroxycinnamoyl-CoA ligase, chalcone synthase, p-hydroxycinnamoyl-CoA:shikimic/quinic acid transferase, caffeoyl-CoA O-methyltransferase, caffeic/5-hydroxyphenolic acid 3,5-O-methyltransferase, sinapic alcohol dehydrogenase, cinnamic alcohol dehydrogenase and glucosyl transferase) in flax seedlings (cv. Nike) treated with non-pathogenic *Fusarium oxysporum* for 3h, 6 h, 12 h, 24 h, 36 h and 48 h.

In this study, flax seedlings were subjected to treatment with a non-pathogenic strain of *F. oxysporum*, and the transcript levels of PR genes, specifically two isoforms of β-1,3-glucanase and chitinase, were investigated. The analyses were conducted at various time points, namely 3, 6, 12, 24, 36, and 48 hours after flax treatment with Fo47. The results are presented in **Figure 2**, which shows a simplified heatmap indicating trends in the observed changes, and **Supplementary Figure 2**, which provides bar charts with detailed statistical analysis.

For the first isoform of β-1,3-glucanase, a notable 2-fold increase in mRNA levels was observed after 12 hours of incubation. However, no significant changes in the transcript level were noted at other time points. On the other hand, the second isoform of β-1,3-glucanase displayed a 1.85-fold increase in mRNA levels after 24 hours of incubation. Interestingly, its expression decreased at 6 hours to 35%, at 36 hours to 20%, and at 48 hours to 12%. Regarding chitinase, a consistent increase in mRNA levels was observed at all time points following incubation with the non-pathogenic strain of *F. oxysporum*. The most substantial increases occurred at 12 and 24 hours of incubation, with a 6.4-fold and 5.8-fold increase, respectively.

### 3.3 Analysis of cell wall components in flax seedlings treatment with a non-pathogenic strain of *Fusarium oxysporum*

**Figure 3:**
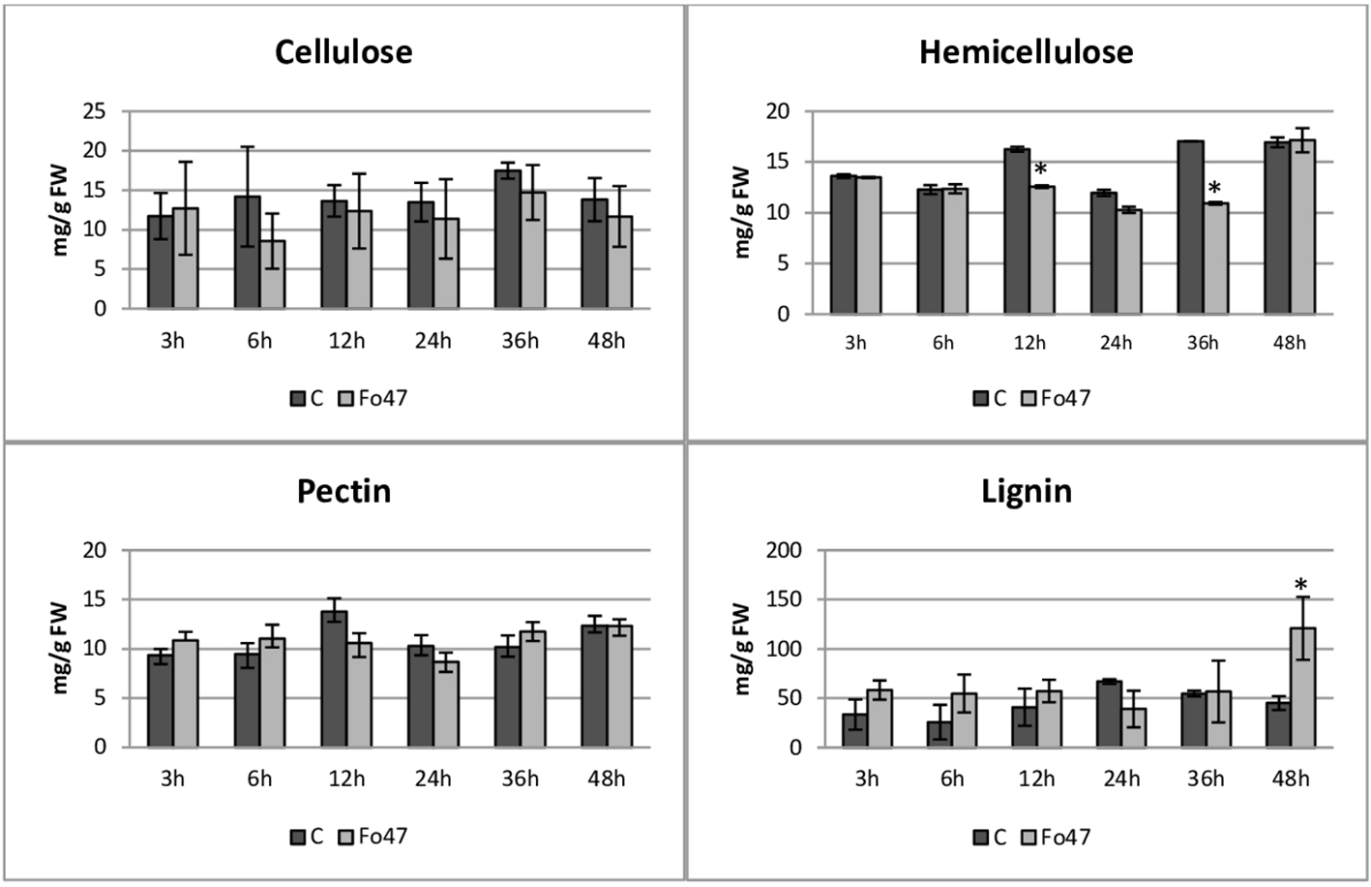
Content of cell wall components in flax seedlings in response to a non-pathogenic strain of *Fusarium oxysporum*. Changes in the content of cellulose, hemicellulose, pectins and lignins in Nike flax seedlings treated with a non-pathogenic strain of *Fusarium oxysporum* (Fo47) at 3 h, 6 h, 12 h, 24 h, 36 h and 48 h compared to control (C). Results are presented as mean ± SD (n=3). The Student’s t-test was used to determine the statistical significance of the obtained results (* -P < 0.05).

The content of cell wall components (cellulose, hemicellulose, pectin and lignin) in flax treated with the non-pathogenic strain of *F. oxysporum* and in the control flax was determined at all time points (3h, 6h, 12h, 24h, 36h and 48h). The cellulose content in flax seedlings remains constant at 13 mg/g FW after treatment with the non-pathogenic strain of *F. oxysporum*, showing no significant changes when compared to untreated seedlings across all analyzed time points (**Figure 3**).

**Figure 4:**
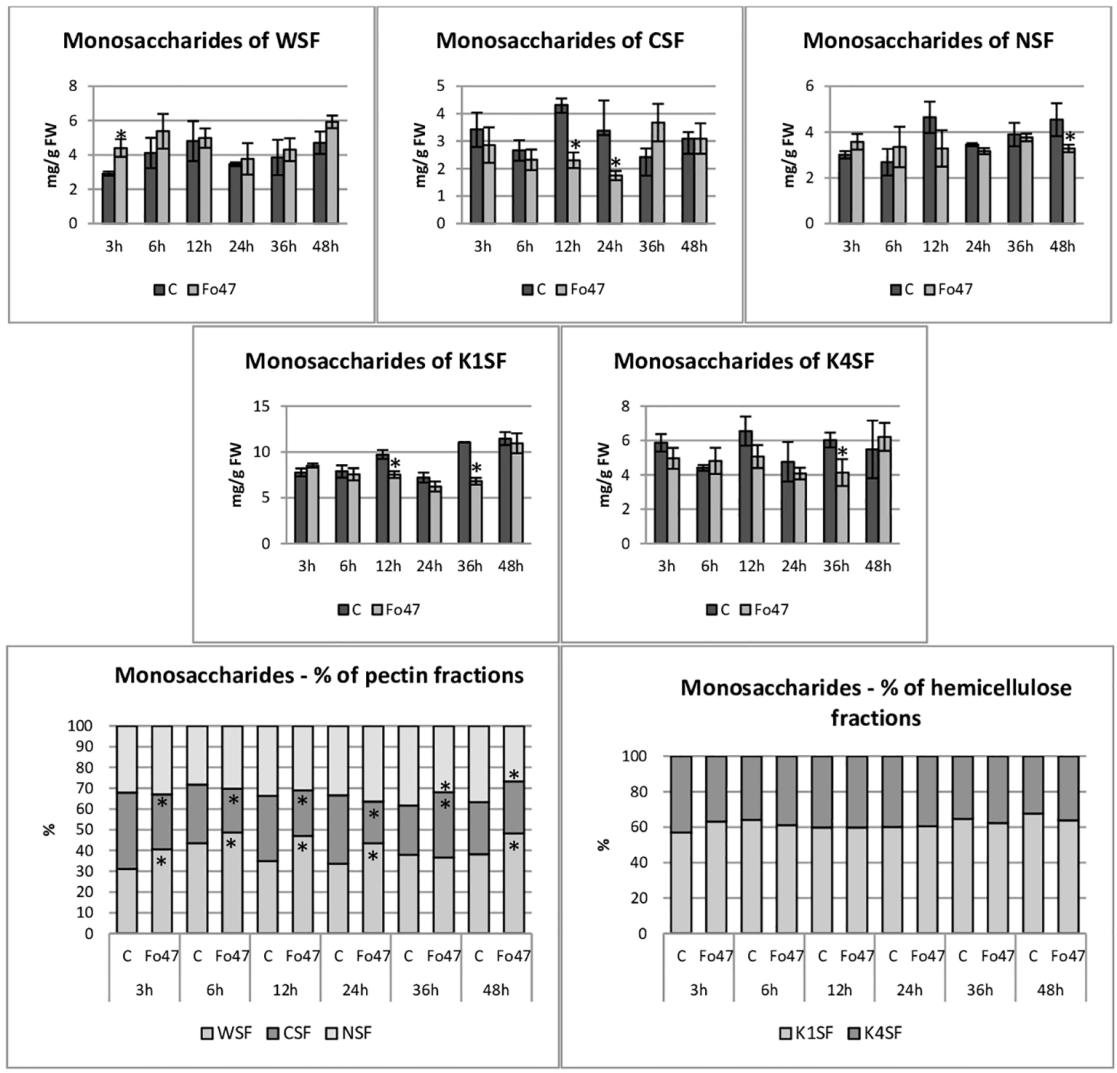
Content of monosaccharides in flax seedlings in response to a non-pathogenic strain of *Fusarium oxysporum*. Changes in the content of monosaccharides and their percentage share in hemicellulose fractions of the cell wall (K1SF - fraction soluble in 1M KOH and K4SF - fraction soluble in 4M KOH) and in pectin fractions of the cell wall (WSF - fraction soluble in water, CSF - fraction soluble in CDTA, NSF – fraction soluble in Na_2_CO_3_) in Nike flax seedlings treated with non-pathogenic strain *Fusarium oxysporum* (Fo47) at 3 h, 6 h, 12 h, 24 h, 36 h and 48 h compared to control (C). Results are presented as mean ± SD (n=3). The Student’s t-test was used to determine the statistical significance of the obtained results (* -P < 0.05).

**Figure 5:**
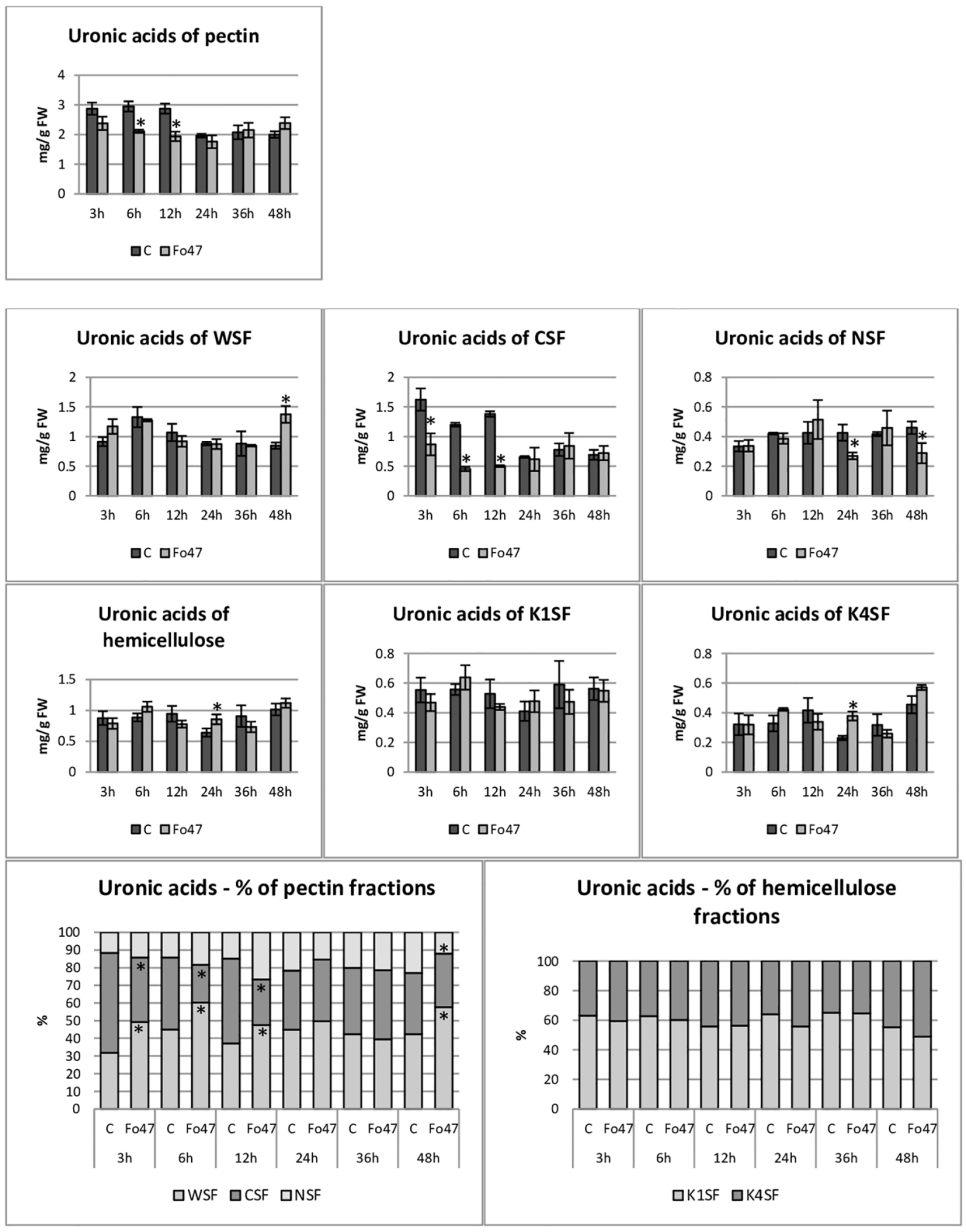
Content of uronic acids in flax seedlings in response to the action of a non-pathogenic strain of *Fusarium oxysporum*. Changes in the content of uronic acids and their percentage share in hemicellulose fractions of the cell wall (K1SF - fraction soluble in 1M KOH and K4SF - fraction soluble in 4M KOH) and in pectin fractions of the cell wall (WSF - fraction soluble in water, CSF - fraction soluble in CDTA, NSF – fraction soluble in Na_2_CO_3_) in Nike flax seedlings treated with non-pathogenic strain *Fusarium oxysporum* (Fo47) at 3 h, 6 h, 12 h, 24 h, 36 h and 48 h compared to control (C). Results are presented as mean ± SD (n=3). The Student’s t-test was used to determine the statistical significance of the obtained results (* -P < 0.05).

The total hemicellulose content (**Figure 3**) was determined as the sum of monosaccharides in hemicellulose fractions of the cell wall (K1SF – fraction soluble in 1M KOH and K4SF – fraction soluble in 4M KOH) (**Figure 4**). In addition, the amounts of uronic acids in the analyzed hemicellulose fractions and their sum are shown (**Figure 5**). The content of hemicellulose in flax seedlings after treatment with non-pathogenic *F. oxysporum* was 10-17 mg/g FW. Reduced hemicellulose content in flax treated with the non-pathogenic strain was observed at 12 and 36 hours of incubation, by 23% and 36%, respectively. It resulted from the reduced content of monosaccharides in K1SF and K4SF in these hours. A slight 1.3-fold increase in the content of uronic acids in hemicellulose was observed 24 hours after treatment with non-pathogenic *F. oxysporum*. It was a consequence of a 1.6-fold increase in the amount of uronic acids in K4SF. Analyzes of the percentage of both monosaccharides and uronic acids in both hemicellulose fractions did not show significant differences in flax treated with Fo47.

The total pectin content (**Figure 3**) was determined as the sum of monosaccharides in the pectin fractions of the cell wall (WSF - water soluble fraction, CSF - CDTA soluble fraction, NSF - Na_2_CO_3_ soluble fraction) (**Figure 4**). Their content did not change significantly in treated flax and control flax during incubation and it was 12.3 mg/g FW after 48 hours of treatment. The analysis of individual fractions showed a 1.5-fold increase in monosaccharide content in WSF after 3 hours of Fo47 treatment and a decrease in monosaccharide content by about 50% at 12 and 24 hours in CSF and by about 30% at 48 hours in NSF. The particular percentage of the simple sugars in the pectin fractions changed over time. From 3 to 24 hours, an increase in monosaccharide content in WSF and a decrease in CSF in flax seedlings treated with Fo47 are noticeable. At the next analyzed time point (36h), we observe an increase in the content of monosaccharides in CSF and a decrease in NSF, while after 48 hours after treatment with non-pathogenic *F. oxysporum*, an increase in monosaccharide content in WSF and a decrease in NSF are observed.

The content of uronic acids was also determined in the pectin fractions of the cell wall (**Figure 5**). In flax treated with non-pathogenic strain of *F. oxysporum*, the content of uronic acids in CSF was reduced by about 50% at 3, 6 and 12 hours of incubation compared to control flax. A smaller amount was also in NSF at 24 and 48 hours of incubation. On the other hand, in the WSF, in the 48th hour of incubation, the content of uronic acids increased 1.6 times. This translated into approx. 30% lower total uronic acid content in pectin at 6 and 12 hours in flax treated with Fo47. Interestingly, the percentage share of uronic acids in the pectin fractions also changed. Already 3 hours after the treatment of flax seedlings with non-pathogenic *F. oxysporum*, a higher content of uronic acids in WSF and a lower content in CSF can be seen. Also after 6 and 12 hours a similar change is noticeable. In the next two analyzed times, there were no changes in the percentage share of uronic acids in pectin fractions. However, after 48 hours of incubation in flax seedlings treated with non-pathogenic *F. oxysporum*, we observed a higher content of uronic acids in WSF and a lower content of uronic acids in NSF.

In flax seedlings treated with the non-pathogenic strain of *F. oxysporum*, the average lignin content at the analyzed time points is measured at 49 mg/g FW (**Figure 3**). However, after 48 hours of treatment, the lignin content significantly increases by 2.7 times compared to the untreated seedlings, reaching a value of 120 mg/g FW.

### 3.4 Infrared spectroscopy and X-ray analysis of cell wall polymers in flax seedlings treatment with a non-pathogenic strain of *Fusarium oxysporum*

There are two main areas of FT-IR spectra that showed significant changes and these are the ranges from 1150 to 1740 cm-1 and from 2800 to 3500 cm-1. Individual bands of FT-IR spectra of flax seedling samples and their assignment are presented in **Table 1**.

**Table 1:** Individual bands of FT-IR spectra of flax seedling samples and their assignment.

Wavelenght (cm ^-1^)	Assignment	References
3500	Lignin, Cellulose, Hemicellulose: O(2)H. . .O(6) intramolecular hydrogen bonds	(Oh et al., 2005; Popescu et al., 2007)
3395	Lignin, Cellulose, Hemicellulose: O(3)H. . .O(5) intramolecular hydrogen bonds	(Oh et al., 2005; Popescu et al., 2007)
3294	Lignin, Cellulose, Hemicellulose: O(6)H. . .O(3) intermolecular hydrogen bonds	(Oh et al., 2005; Popescu et al., 2007)
1740	Hemicellulose, Pectin: C = O stretching in unconjugated ketone, carbonyl, and aliphatic groups, C = O stretching in ester carbonyl	(Szymanska-Chargot and Zdunek, 2013; Largo-Gosens et al., 2014; Lahlali et al., 2017; Chow and Ting, 2019; Dinant et al., 2019; Javier-Astete et al., 2021)
1655	Lignin: absorbed O-H and conjugated C-O	(Chow and Ting, 2019)
1600	Lignin: aromatic ring vibration Pectin: COO- antisymmetric stretching	(Popescu et al., 2007; Szymanska-Chargot and Zdunek, 2013; Largo-Gosens et al., 2014; Cheng et al., 2016; Dinant et al., 2019; Javier-Astete et al., 2021)
1455	Lignin: C = O stretching and aromatic skeletal vibration. Cellulose, Hemicellulose, Pectin: CH and OH in-plane bending	(Popescu et al., 2007; Cheng et al., 2016; Javier-Astete et al., 2021)
1372	Cellulose and hemicellulose: CH bending, C–H deformation vibration	(Popescu et al., 2007; Szymanska-Chargot and Zdunek, 2013; Largo-Gosens et al., 2014; Cheng et al., 2016; Chow and Ting, 2019; Dinant et al., 2019; Javier-Astete et al., 2021)
1343	Cellulose: CH bending Lignin: C_1_–O vibrations in syringyl derivatives	(Popescu et al., 2007; Cheng et al., 2016)
1319	Cellulose: CH2 wagging	(Popescu et al., 2007; Szymanska-Chargot and Zdunek, 2013; Largo-Gosens et al., 2014; Cheng et al., 2016; Javier-Astete et al., 2021)
1260	Lignin: Guaiacyl ring breathing, C–O linkage in guaiacyl aromatic methoxyl groups	(Popescu et al., 2007; Largo-Gosens et al., 2014; Cheng et al., 2016; Dinant et al., 2019)
1230	Lignin: syringyl ring breathing, Hemicellulose, pectin: C–O stretching	(Popescu et al., 2007; Szymanska-Chargot and Zdunek, 2013; Lahlali et al., 2017; Dinant et al., 2019)
1150	Cellulose, hemicellulose, pectin: C–O–C asymmetric stretching	(Popescu et al., 2007; Szymanska-Chargot and Zdunek, 2013; Largo-Gosens et al., 2014; Escudero et al., 2017; Lahlali et al., 2017; Dinant et al., 2019; Javier-Astete et al., 2021)

The obtained results show changes in spectra resulting from dynamic biological processes, including plant development and colonization with a non-pathogenic strain of *F. oxysporum*. To show changes resulting from fungus penetration of plant tissue, the ratio of the spectra of infected to uninfected plants was calculated.

**Figure 6:**
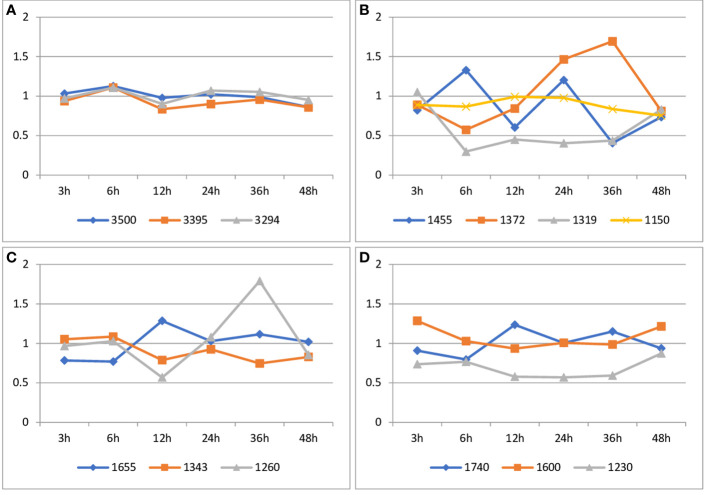
Analysis of the integral intensities of bands in FT-IR spectra of flax seedlings infected with non-pathogenic *Fusarium oxysporum* compared to non-infected flax seedlings. **(A)** Changes in the integral intensities of bands at 3500 cm^-1^, 3395 cm^-1^, 3294 cm^-1^. **(B)** Changes in the integral intensities of bands at 1455 cm^-1^, 1372 cm^-1^, 1343 cm^-1^ and 1319 cm^-1^ and 1150 cm^-1^. **(C)**. Changes in the integral intensities of bands at 1655 cm^-1^ and 1260 cm^-1^. **(D)**. Changes in the integral intensities of bands at 1740 cm^-1^, and 1600 cm^-1^ and 1230 cm^-1^.

In the FT-IR spectra, the broad absorption bands observed in the range 3650-3000 cm^-1^ are defined as bands connected with hydroxyl groups engaged in forming hydrogen bonds (intramolecular and intermolecular) in cellulose, hemicellulose and lignin (**Table 1**). The values of the intensity integral bands characteristic of hydrogen bond stretching vibrations do not differ statistically significantly for flax seedlings treated with Fo47 compared to untreated seedlings (**Figure 6A**; **Supplementary Figure 3**).

The values of the integral intensity of the band at approximately 1150 cm-1 are lower for flax seedlings treated with Fo47 after 3 hours (by 10%), 6 hours (by 10%), 36 hours (by 15%) and 48 hours (by 25%). Treatment of flax seedlings with a non-pathogenic strain of *F. oxysporum* resulted in a decrease of the integral intensities for this band of 1319 cm-1 after 6 hours (by 70%), 12 hours (55%), 24 hours (60%) and 36 hours (25%). Treatment of plants with Fo47 also resulted in a decrease in the integral intensity for the 1372 cm-1 band after 6 h (by 43%), 12 h (15%) and 48 h (20%), while a 1.5-fold increase after 24 h and 1.7 -fold increase 48 hours. In the case of the 1455 cm-1 band, the observed decrease in the integral intensity for this band after 3 h (by 20%), 12 h (by 40%), 36 h (by 60%) and 48 h (by 25%) and 1.3-fold increase at 6 h and 1.2-fold increase at 24 h after treatment with nonpathogenic *Fusarium oxysporum* (**Figure 6B**; **Supplementary Figure 3**).

The values of the integral intensities of the 1343 cm-1 band are lower for flax seedlings treated with Fo47 after 12 h (by 20%), 36 h (by 25%) and 48 h (by 17%). However, the values of the integral intensity of the 1260 cm-1 band are lower for flax seedlings treated with Fo47 after 12 h (by 43%) and 1.8-fold higher after 36 h. The intensity values of integral bands at 1655 cm-1 do not change significantly in flax seeds treated with Fo47 (**Figure 6C**; **Supplementary Figure 3**).

The total intensity of the 1740 cm-1 band has lower values for flax seedlings treated with the non-pathogenic *Fusarium oxysporum* after 3, 6 and 48 hours, and higher values after 12 and 36 hours. However, the intensity values of integral bands at 1600 cm-1 do not change significantly in flax seeds treated with Fo47. The values of integral band intensities at approximately 1230 cm-1 are lower by 25% after 3 hours and by 40% after 12, 24 and 36 hours for seedlings treated with the non-pathogenic strain compared to untreated seedlings (**Figure 6D**; **Supplementary Figure 3**).

FT-IR spectra measured for the studied flax seedlings were analyzed using the PCA procedure in order to visualize the main trends of the spectroscopic data. This approach applied to the bands characteristic for the cellulose shows that the diagnostic meaning for this component has a band at 1319 cm^-1^. The PC1 for these data is 89.67% and PC2 – 7.68%. It means that the band chosen among other pairs of bands is the best fit for the cellulose content in the flax seedlings. The PCA analysis applied to the other IR bands observed for the flax seedlings shows that the band at 1343 cm^-1^ has a diagnostic value for the lignin content, whereas those of 1600 cm^-1^ should be used for the pectin. For the former band, the PC1 takes the value of 91.74% and PC2 – 7.63% and for the second band PC1 is 91.59% and PC2 7.64%.

We determined the crystallinity index (CI), which serves as an indicator of the relative amount of crystalline material in cellulose (**Table 2**). A 13% decrease in CI was observed in flax seedlings after 3 hours after Fo47 treatment and a 1.12-fold increase after 6 h, a 1.15-fold increase after 12 h, a 1.2-fold increase after 24 h and a 1.1-fold increase after 36 h. This suggest that the cellulose structure of flax seedlings changes as a result of the infection. Moreover, the crystallinity index increases faster in the case of infected seedlings compared to control seedlings: from approx. 20% for flax seedlings treated with Fo47 after 3 h to 58% for seedlings treated with Fo47 after 48 h and from approx. 23% for untreated seedlings after 3 h to 56% after 48 hours.

**Table 2:** The crystallinity index (CI) of flax seedlings infected with non-pathogenic *Fusarium oxysporum* (Fo47) at 3 h, 6 h, 12 h, 24 h, 36 h and 48 h compared to control (C).

	3h	6h	12h	24h	36h	48h
**C**	23	25	39	40	49	56
**Fo47**	20	28	45	48	53	58

### 3.5 Transcript levels of cellulose metabolism genes in flax seedlings treatment with a non-pathogenic strain of *Fusarium oxysporum*

The results of the analysis of mRNA levels of cellulose synthesis and degradation genes in flax incubated with a non-pathogenic strain of *Fusarium oxysporum* compared to control flax are shown in **Figure 2** (simplified heatmap showing trends in observed changes) and **Supplementary Figure 4** (bar charts with full statistical analysis).

The highest increase in transcript level was observed for the cellulose synthase 3 gene (from a 3.2-fold increase in 3 h to a 2.5-fold increase in 12 h), a lower but also statistically significant increase for the cellulose synthase 1 gene (1.5-fold at 12 h and 1.7-fold at 36 h) and for cellulose synthase 4 and 5 genes, where transcript levels increased only at one time point (2-fold increase in CSL4 expression at 12 h and 2.4 -fold increase in CSL5 expression at 48 h). In contrast, the CSL2 mRNA level was reduced (up to 80% at 6 h, 67% at 24 h and up to 77% at 48 h). Cellulase 2 was characterized by a 1.9-fold increase in mRNA level at 6 h of incubation with a non-pathogenic strain of *F. oxysporum*, while cellulase 1 was characterized by a 1.65-fold increase in mRNA level at 3 h and a decrease to approximately 75% at 24, 36 and 48 hours.

### 3.6 Transcript levels of hemicellulose metabolism genes in flax seedlings treatment with a non-pathogenic strain of *Fusarium oxysporum*

Analysis of mRNA levels of genes participating in hemicellulose synthesis [glucomannan 4-β-mannosyltransferase (GMT), galactomannan galactosyltransferase (GGT), xyloglucan:xyloglucosyl transferase (XXT)] and hemicellulose degradation [endo-1,4-β-xylanase (XYN), 1,4-α-xylosidase (XYLa), 1,4-β-xylosidase (XYLb), α-galactosidase (GS), endo-β-mannosidase (MS), β-glycosidase (GLS)] in flax seedlings incubated with the non-pathogenic *F. oxysporum* for 48 and analyzed at 3 h, 6 h, 12 h, 24 h, 36 h, 48 h is presented in **Figure 2** (heatmap) and **Supplementary Figure 5** (bar charts with full statistical analysis).

In the levels of GMT and GGT gene transcripts a significant decrease (up to about 50-30%) was observed from 6 to 48 hours of incubation with a non-pathogenic strain of *F. oxysporum*. In the third hemicellulose synthesis gene, XXT, the mRNA level was reduced to about 65% at 6, 12 and 36 hours.

Analysis of the mRNA levels of genes involved in hemicellulose degradation in flax after treatment with non-pathogenic *F. oxysporum* showed a decrease in the level of XYN mRNA to 50% (from 12 to 36 hours), a decrease in the level of XYLb mRNA to about 50% (in 6 hours and from 24 to 48 hours), a 1.45-fold and 2.4-fold increase in GLS gene transcript levels (at 6 and 48 hours, respectively) and a 1.4-1.8-fold increase in XYLa mRNA levels (at 6, 12 and 36 hours). The highest changes in the transcript level were found in the GS gene, whose mRNA level increased during incubation (from a 1.6-fold increase in 3 hours to a 5.8-fold increase in 36 hours).

### 3.7 Transcript levels of pectin metabolism genes in flax seedlings treatment with a non-pathogenic strain of *Fusarium oxysporum*

Analysis of mRNA levels of the genes of pectin synthesis [UDP-D-glucuronate 4-epimerase (GEA), galacturonate transferase 1 (GAUT1), galacturonate transferase 7 (GAUT7), xylose:rhamnogalacturonate transferase II (RGXT), arabinose transferase (ARAD), pectin methyltransferase (PMT)] and degradation (pectin methylesterase 1 (PME1), pectin methylesterase 3 (PME3), pectin methylesterase 5 (PME5), polygalacturonase (PG), pectin lyase (PaL), pectate lyase II (PL)) in flax seedlings incubated with the non-pathogenic F. oxysporum for 3 h, 6 h, 12 h, 24 h, 36 h and 48 h is presented in **Figure 2** (heatmap) and in **Supplementary Figure 6** (bar charts with full statistical analysis).

Changes in the transcripts levels of both pectin synthesis and degradation genes were observed in the flax treated with non-pathogenic strain of *F. oxysporum*, with the difference that the mRNA levels of most pectin synthesis genes was reduced, and the mRNA levels of degradation genes showed quite a large variation between individual genes. The transcript level was reduced in the pectin synthesis genes: GAE (up to about 60% in 3, 6, 24 and 36 hours of incubation), GAU1 (up to 75% in 36 hours), GAU7 (up to 75% in 6, 24 and 36 hours), RGXT (up to 60% in 36 hours), ARAD (up to 70% in 24 hours and 50% in 36 hours) and PMT (up to 60% in 36 hours). In addition, a slight 1.3-fold increase in mRNA levels was observed for the RGXT gene at 48 hours and a 1.9-fold increase for the PMT gene at 3 hours of incubation with non-pathogenic *F. oxysporum*.

PME1 transcript levels were reduced to 55% only at 48 hours, while PME3 transcript levels were reduced to 12-40% between 6 and 48 hours and increased 1.5-fold at 3 hours of incubation with non-pathogenic strain of *F. oxysporum*. In PME5, the mRNA level increased 1.7-fold in 3 hours, and 1.6-fold in 12 hours, while it decreased to 70% in 24 hours. The PG gene showed a similar pattern of changes in mRNA levels as PME3, revealing a 1.5-fold increase in 3 h. and a drop to 55% in 12 hours. and up to 30% at 36 hours. Pectin lyase showed a 60-40% decrease in mRNA levels at 12, 36 and 48 hours and pectate lyase a 1.4-2-fold increase mRNA level from 3 to 48 hours.

### 3.8 Transcript levels of lignin metabolism genes in flax seedlings treatment with a non-pathogenic strain of *Fusarium oxysporum*

Changes in the expression of genes of lignin metabolism [phenylalanine ammonia lyase (PAL), 4-hydroxycinnamyl-CoA ligase (4CL), chalcone synthase (CHS), p-hydroxycinnamyl-CoA:shikimic acid transferase (HCT), caffeoyl-CoA O-methyltransferase (CCoACMT), caffeic acid/5-hydroxyphenolic acid 3,5-O-methyltransferase (COMT), sinapic alcohol dehydrogenase (SAD), hydroxycinnamic acid dehydrogenase (CAD) and glucosyltransferase (GT)], a part of the phenylpropanoid pathway in the flax seedlings exposed to non-pathogenic *F. oxysporum* for 48 h, are presented in **Figure 2** (heatmap) and **Supplementary Figure 7** (bar charts with full statistical analysis).

For most of the genes (PAL, HCT, CCOAOMT, COMT and SAD) a similar pattern of mRNA levels was observed in flax treated with non-pathogenic strain of *F. oxysporum*. Transcript levels of these genes increased from 3 hours of incubation (1.4 to 1.7-fold increase in individual genes), peaked at 12 hours (2.5 to 4.5-fold increase in expression), and then decreased and equal the level of mRNA before incubation or even decreased to 40-70% at 36 and/or 48 hours. The remaining lignin metabolism genes differed significantly from the mRNA levels profile presented above. An increase in transcript level was noted for the 4CL gene (approximately 1.6-fold increase at 3, 12 and 24 h), CAD (2-fold at 3 hours, 2.5-fold at 24 hours, 1.4-fold in 36 hours and 1.9-fold in 48 hours) and GT (3.5-fold in 3 hours, 8.7-fold in 24 hours, 4.2-fold in 36 hours and 2. 9 times in 48 hours). In addition, a 2.5-fold increase in mRNA levels at 3 hours was characteristic of the CHS gene, whose transcript levels at 36 and 48 hours were reduced to 50% and 40%, respectively.

## 4 Discussion

The cell wall serves as the first protective barrier in plants against pathogenic infections. What role does it play in the penetration of non-pathogenic strains into plants, which are intended to prepare the plants for defense against future infections? How does the composition and structure of the cell wall polymers change, and which genes responsible for the metabolism of these polymers are involved in the plant’s response to the entry of non-pathogenic strains? We wanted to answer these questions by conducting analyses on flax seedlings treated with a non-pathogenic strain of *F. oxysporum*.

Our aim was to observe the response of flax seedlings treated with Fo47 over time, therefore we performed the analyzes 3, 6, 12, 24, 36 and 48 hours after treatment. Microscopic observations indicated the presence of mycelial hyphae in the plants 6 hours after treatment, while murein transglycosidase gene of the fungus was detected already at 3 hours. This may indicate a higher sensitivity of the q-PCR method compared to microscopic observations. In the following hours, an increase in the number of mycelial hyphae and an increase in the level of mRNA of murein transglycosidase gene in plants were observed. Both methods confirm the rapid penetration and spread of the fungus in the plant. Microscopic observations conducted by Martínez-Soto illustrating the course of colonization of tomato plants by the Fo47 endophyte showed that at 1 dpi, Fo47 penetrated the tomato epidermal tissue, and then colonized the root cortex at 2 and 3 dpi, and the root vascular system of lateral roots was colonized at 4 dpi (Martínez-Soto et al., 2023). Similar relationships for the colonization of tomato plants by Fo47 were noticed by Olivain and Alabouvette, in which Fo47 colonized the root surface within 24 hours and the root tip after 48 hours. After 48 h, several hyphae were also observed penetrating the epidermis, leading to internal colonization of the root cortex (Olivain and Alabouvette, 1997). However, research by Bolwerk and others showed that colonization of the tomato root surface by Fo47 was observed after 4 days and strongly increased on days six and seven (Bolwerk et al., 2005). Fo47 colonization was usually restricted to the crown level, and the fungus was rarely observed at cotyledon level (Constantin et al., 2020). The rapid colonization of flax seedlings by Fo47 compared to other research teams may be due to a different model of treating plants with an endophyte and constant exposure to Fo47. Moreover, plants were treated from day 12 after germination and the treatment continued for 2 days, which means that the plant was treated and analyzed while developing the cotyledon, which also may have an impact on plant colonization. Additionally, our microscopic observations show that Fo47 has the ability to migrate into the stem, which has not been observed before. The ability to migrate to the stem of *Lotus japonicus* is demonstrated by the non-pathogenic *Fusarium solani* strain FsK, whose hyphae multiply in the plant’s vascular bundle (Skiada et al., 2019).

The defense reaction of flax plants against the non-pathogenic *F. oxysporum* was triggered already 3 hours after treatment, which is evidenced by the increase in the transcript level of the chitinase gene at that time. This does not have to be related to the presence of the fungus in plants, because the increase in PR gene expression may result from the action of fungal elicitors. Moreover, an increase in chitinase mRNA was observed at all analyzed hours, while an increase in glucanase isoform transcripts was only noticeable at 12 and 24 hours. Similar changes were observed in tomato plants after inoculation with Fo47, where an increase in extracellular chitinase (CHI3) was observed in the roots after 48 h, and an increase in intracellular chitinase (CHI9), CHI3 and intracellular β-1,3-glucanase (GLUB) after 72 h. and after 96 h extracellular β-1,3-glucanase (GLUA) and GLUB. In tomato cotyledons after inoculation of Fo47, GLUA gene was upregulated after 72 hpi and GLUA, GLUB, CHI3 I CHI9 after 96hpi (Aimé et al., 2013). Also, transcriptome analysis of soybean roots to colonization with the root endophytic fungus *Piriformospora indica* revealed that two chitinase-like proteins were highly upregulated (Bajaj et al., 2018). The effect of elicitors and the endophytic fungus *Gilmaniella* sp on *Atractylodes lancea* plants was checked by Wang et al., showing that both fungus and elicitor enhanced defense-related enzyme activities, with the chitinase activity being the highest and reaching a maximum at 10 dpi, while the β-1,3- glucanase activity was apparently enhanced in the first 20 days. In elicitor-treated groups, however, most of the enzymes were activated during the early stage, and their highest levels were generally lower than those of the fungus-inoculated groups (Wang et al., 2012). Also strain Fo47 induces resistance in tomato plants against *Fusarium* through increased chitinase, β-1,3-glucanase, and β-1,4-glucosidase activity in plants (Fuchs et al., 1997). Similarly, treatment of *Withania somnifera* plants with endophytic bacteria *Bacillus amyloliquefaciens* and *Pseudomonas fluorescens* increased the expression level of PR genes, showed maximum upregulation for β-1,3-glucanase and chitinase in treated plants at 72 hpi (Mishra et al., 2018). Also, transcriptome analysis of rice roots colonized by endophyte *P. indica* revealed the increase of chitinase (Chi4) gene expression (Nivedita et al., 2020).

Spectrophotometric determination showed no changes in cellulose content in flax seedlings after Fo47 treatment. However, the IR analysis indicates possible changes in bonds among the cell wall polymers. The cellulose crystallinity index changes over time: in the initial hours of flax’s response to Fo47, its decrease is observed, then it increases and in the last analyzed hour it does not change compared to the control. This suggests that the structure of cellulose is constantly rearranged, taking on a less ordered form with greater activity, and then subsequently a more ordered form with lower activity.

Transcript analysis shows a significant increase in the mRNA level of the cellulose synthase 3 gene at the initial times (from 3 to 12 h) of the plant response to Fo47 treatment. In the case of infection of flax seedlings with the pathogenic strain of *F. oxysporum*, the mRNA level of this gene did not change (Wojtasik et al., 2016). The remaining isoforms of the cellulose synatase gene change slightly, which also distinguishes the response of flax to Fo47 and pathogenic *F. oxysporum*, where reduced levels of most isoforms of this gene were observed. In the case of cellulases, the transcript level of both isoforms initially increases (at 3 and 6 h) and then decreases (from 12 h). Similar changes were observed in seedlings treated with pathogenic *F. oxysporum*.

During the response of flax seedlings to treatment with non-pathogenic *F. oxysporum*, a decrease in the hemicellulose content was noticeable at 12 and 36 hours, resulting from lower monosaccharide content. This could be a consequence of a significant decrease in the mRNA level of genes involved in hemicellulose biosynthesis at all analyzed time points. Similarly, the down-regulated xyloglucan endotransglucosylase (XTH3) gene involved in hemicellulose rearrangement was observed in both pepper and tomato after Fo47 induction (Veloso and Díaz, 2021). However, transcriptome analysis of soybean roots to colonization with the root endophytic fungus *P. indica* reveals upregulated genes with potential roles in cell wall remodeling included a xyloglucan endotransglucosylase/hydrolase 32 family gene (Bajaj et al., 2018). Among the analyzed hemicellulose degradation genes, two: endo-1,4-β-xylanase and 1,4-β-xylosidase were characterized by a decrease in mRNA levels, and one: α-galactosidase - a significant increase. The remaining ones did not show any significant changes. The pathogenic strain caused similar changes in flax seedlings: it reduced the hemicellulose content, most likely by a decrease in the mRNA of hemicellulose synthesis genes, and caused similar changes in the transcript levels of hemicellulose degradation genes. The only gene differentiating both of these strains was the β-glycosidase gene, the mRNA level of which did not change significantly after treatment with non-pathogenic Fo47, while after treatment with pathogenic Fol it was significantly reduced. Worth mentioning is the α-galactosidase gene, which changes similarly after treatment with both strains, showing a significant increase in mRNA levels over time (Wojtasik et al., 2016). This enzyme is responsible for cutting off galactose from galactomannan (Malgas et al., 2015). There is a report that enzyme that degrade hemicelluloses in the cell wall and release galactose can directly contribute to the alteration in cellulose crystallinity during secondary cell wall synthesis (Roach et al., 2011). The hypothesis that galactosidase, by cleaving galactose from galactomannan, indirectly enabled the formation of hydrogen bonds in cellulose and thus led to an increase in its crystallinity in flax seedlings in response to treatment with a non-pathogenic strain of *F. oxysporum*, requires further research and confirmation. Treatment of flax seedlings with a non-pathogenic strain of *F. oxysporum* did not cause changes in the total pectin content. However, the analysis of monosaccharide content showed an increase in the % of WSF and a decrease in CSF from 3 to 24 h, followed by an increase in the % of CSF and a decrease in NSF in 36 h and an increase in WSF and a decrease in NSF in 48 h. Also, an increase in the percentage of uronic acids in WSF and a decrease in CSF from 3 to 12 h was observed. Changes in the percentage of monosaccharides in pectin could result from rearrangements of the structure of these polymers, which also occurs during pathogenic infections. Additionally, IR analysis (at 1740 cm^-1^) revealed a dynamic change in the level of methylesterification of carbroxyl groups of pectin in flax seedlings treated with Fo47 over time. At 3 and 6 h a decrease in the level of methylesterification of carbroxyl groups was observed, while at 12, 36 and 48 h an increase was observed. This condition is also typical during a pathogenic infection, because removal of the methyl group from homogalacturonan by pectin methylesterase, resulting in loosening of cell wall structures, enabling pectin degradation by polygalacturonases and pectin lyases, and also cellulose and hemicellulose by cellulases and hemicellulases (Willats et al., 2001). Transcript levels of the PME 1 and PME 3 genes were significantly reduced after treatment with the non-pathogenic strain 6 to 48 h, in contrast to flax seedlings treated with the pathogenic strain, where an increase in the mRNA of these genes was observed. Most likely, this may be due to the expression at different levels of fungal PME genes that are activated during infection. Analysis of pectin degradation genes showed a decrease in the level of polygalacturonase and pectin lyase mRNA, and an increase in the amount of pectate lyase gene transcript, which distinguishes the response of plants to strains of different pathogenicity. However, treatment of flax seedlings with Fo47, similarly to the treatment with pathogenic *F. oxysporum*, resulted in a reduction in the level of transcripts of genes involved in pectin synthesis (Wojtasik et al., 2016). Chow’s research showed that endophytic fungi (*Diaporthe phaseolorum*, *Trichoderma asperellum*, *Penicillium citrinum*) and pathogenic fungus (*Ganoderma boninense*) in a similar way degrade the plant cell wall of oil palm and change its structure by secreting cell wall degradative enzymes (CWDEs) such as cellulase, laccase, pectinase, and xylanase, thereby infiltrating, colonizing and proliferating tissues in plant. However, most endophytes do not break down lignin and carbohydrates, resulting in nonpathogenic and asymptomatic infection by the endophytes (Chow and Ting, 2019).

Our research shows that treatment of flax seedlings with the non-pathogenic *F. oxysporum* led to changes in lignin content. Spectrophotometric analysis showed a significant increase in lignin 48 h after treatment. Elevated lignin biosynthesis ensues as a consequence of heightened activation of the phenylpropanoid pathway, whereas the initiation of the lignification process is prompted by the presence of abundant reactive oxygen species, particularly during pathogenic infections (Bhuiyan et al., 2009; Collinge, 2009). Increase in expression of genes associated with the phenylpropanoid pathway, as well as those directly involved in lignin synthesis, was discerned in wheat following infection with *Fusarium graminearum* and *Puccinia triticina* (Bi et al., 2011) and in cotton infected with *Verticillium dahliae* (Xu et al., 2011). However, research conducted by Martínez-Soto showed the induction of lignin deposition at early stages in roots of both plant hosts (*A. thaliana* and *Solanum lycopersicum*) colonized by the endophyte Fo47, which was a consequence of up-regulation of the lignin biosynthesis gene (MYB63) (Martínez-Soto et al., 2023). Genes of the phenylpropanoid pathway provide substrates for the production of lignin, but they are also involved in the production of other compounds produced in response to infections. In the case of treating flax seedlings with a non-pathogenic strain, we observe a significant increase in the mRNA levels of these genes already in 3 h, which remains high for up to 24 h, and then for most of these genes it significantly decreases at 36 and 48 h. However, the pathogenic strain of *F. oxysporum* causes a decrease in the mRNA levels of these genes at 6 and partially at 12 hours, and only then their significant increase lasting up to 48 hours. Analyzing the genes directly involved in the synthesis of lignin substrates, it can be seen that already 3 hours after Fo47 treatment, there is a significant increase in the transcript level of these genes, which lasts for up to 24 hours for SAD and then drops significantly, while for CAD it remains at a high level for up to 48 hours. Pathogenic Fol shows a similar expression pattern for CAD, while for SAD we observe an increase in the mRNA level of this gene from 6 h, but it is lower compared to that caused by a non-pathogenic strain, and a decrease only after 48 h. The PAL activity induced by non-pathogenic fungus *Gilmaniella* sp in plants *Atractylodes lancea* increased consistently over a long period of time, and reached a peak in the late stage. Its highest level was markedly greater than that of the elicitor treated groups, and was three fold higher than the level in the control groups (Wang et al., 2012). Also non-pathogenic mutant of *Colletotrichum magna* strains induced resistance in *Citrullus lanatus* and *Cucumis sativus* plants by increase in peroxidase activity, phenylalanine ammonia lyase enzyme, and lignin deposition, which could help protect the plants from infection caused by *C. orbiculare* (Redman et al., 1999). However, the analysis of the transcriptome of soybean roots to colonization with the root endophytic fungus *P. indica* showed that the caffeate o-methyltransferase 1 (COMT1) gene was upregulated and CAD gene was downregulated (Bajaj et al., 2018).

As already indicated, the chemical and genetic analysis of plants, was supplemented with infrared spectroscopy. Due to fungal colonization dynamics (hyphae adhering to and growing on epidermal cells, movement along the stem, complex polymer structure of the cell wall) using, IR spectra of the chemical group characteristic for the polymeric component of the cell wall seems to be appropriate supplementary method. IR is one of the most common, relatively inexpensive optical techniques and, therefore, a widely used as non-invasive technique due to its usefulness in identifying compounds and determining their structure There are wavelengths in the IR spectra than can be attributed to particular polymers (as shown in **Table 1**) and particular bands can indicate various changes in the structure. As spectral analysis was done on whole stems and not isolated fractions it allows to look as a structure as a whole but is better suited to indicate presence or behavior of certain bonds than quantitative analysis of single polymers in the mixture.

Therefore in this article, the focus was on the comparative analysis of vibrations of chemical groups specific to the analyzed polymers, rather than their quantity. In order to approximate the effect of *Fusarium* infection on seedlings on day 12, FT-IR vibration spectra were taken. The obtained results show several discrete changes in spectra resulting from both dynamic biological processes, plant cultivation and fungal infection.

There are two main areas of the spectra that showed significant (over 20%) changes and these are the ranges from 1150 to 1740 cm^-1^ and from 2800 to 3500 cm^-1^. The changes appeared at various stages of plant growth after infection. Detailed analysis showed that in the first hours, reasonable changes were in the C-O and C=O stretching attributed to pectin (1230 and 1740 cm^-1^), a 25% decrease in the vibration of the C-O groups was found. CH2 wagging (1319 cm^-1^) and CH bending of cellulose (1372 cm^-1^) showed a 70% and 43% reduction in vibration, respectively (Cheng et al., 2016) (El-Sakhawy et al., 2018).While the lignin aromatic ring vibration (1600 cm^-1^) showed more than 30% increase, and the in-plane bending of cellulose, lignin and hemicellulose O-H (1455 cm^-1^) increased up to 30% (Cheng et al., 2016). Discrete C=O stretching was also detected for hemicellulose and extractable carbonyl esters (1740 cm^-1^), which showed a 10% decrease in vibration after 3 hours and a 20% decrease in vibration after an additional 3 hours. The region of 3294-3500 cm^-1^ was assigned to intramolecular and intermolecular OH stretching in lignin, cellulose and hemicellulose and no significant changes were observed there (Popescu et al., 2007; Javier-Astete et al., 2021).

During the course of the experiment the levels of the integral intensities at the measured wavelengths changed (even by 60-80%) for the samples treated with Fo47, but returned to those of the control after 48 hours. The obtained data confirmed time-dependent dynamic changes in cell wall components during the infection process.

At the earlier stage of infection, both the IR absorbance of lignin was consistent with the chemical analysis, but at the later stage of infection, IR absorbances are inconsistent with the chemical analysis. The difference can probably be explained by the strong absorbance of CH and OH in-plane bending in all polysaccharide groups (1455 cm^-1^) and the presence of extractives derived from pectin and hemicellulose, which can mask lignin vibrations (Szymanska-Chargot and Zdunek, 2013; Javier-Astete et al., 2021).The primary cell wall consists of a xyloglucan-cellulose skeleton embedded in a matrix of hemicellulose and pectic polysaccharides, the secondary cell wall is formed as a result of lignin deposition. Lignin forms ester bonds through its alcohols with hemicellulose through side residues of 4-O-methyl-d-glucuronic acid glucuronoxylates. Lignin and all other polysaccharides have been found to be acetylated on aliphatic side chains (Sista Kameshwar and Qin, 2018) therefore, IR spectra of such a complex structure do not seem to be a good predictor of the content of a specific polymer. However, IR spectra are certainly useful in identifying compounds and determining their structure (Zhou et al., 2011; Szymanska-Chargot and Zdunek, 2013; Javier-Astete et al., 2021).

In summary, cell wall polymer vibration data show time-dependent changes and, interestingly, they exhibit recovery characteristics. The infrared vibration spectra for all cell wall polymers periodically increase and decrease during infection, with an invariant area in between. The recovery of the spectra probably reflects the transient deformation of the polymer structure due to the penetration of the epidermis by Fusarium. It is quite interesting that when watermelon seedlings were infected with a pathogenic strain of *Fusarium oxysporum*, it was observed that individual hyphae reached the edge of the xylem vessels 6 days after inoculation and by that time the seedlings still appeared healthy and symptom-free when the first visible histological changes were observed 2 days after vaccination (Zhang et al., 2015).

Defensive responses of the host flax to the non-pathogenic *F. oxysporum* appeared as early as 3 hours after inoculation. All cell wall polymers react by changing the vibration intensity of a specific chemical group. Changes in the intensity of vibrations of stretching and bending bonds within these groups undoubtedly suggest a rearrangement of the polymer structure. It is difficult to provide any model for this rearrangement as it would be a challenge for further research. The difference in the vibration intensity of the chemical group between the first and last day of infection is probably related to the penetration of different organs as the hyphae move along the plant. Different flax organs have characteristic cell wall contents, proportions of pectic components and cross-linking glycans, and individual polymers. For example, xylan and xyloglucan in the root and hypocotyl exist in a mol% base-soluble fraction of 55/20 and 45/30, respectively (Gorshkova et al., 1996).

Some data suggest that changes in the absorption spectra of cell wall polymers may reflect the availability of compounds. For example, more monosaccharides can be found in the aqueous fraction of pectin from extracted infected plants. Also, the increased availability of xylene cell wall components and its conversion to furfural may be responsible for the overestimation of lignin content measured spectrophotometrically.

Based on the IR spectra, it is suggested that perhaps each stage of the early phase of infection, such as attachment to the epidermal cell layer, germination of spores, spread of hyphae, formation of mycelium until reaching the xylem vessels, may be reflected in changes in the absorption of chemical groups of the spectrum. What is fascinating is that the structure of the polymers is restored to its original state after the *Fusarium* spreads along the stem and passes through the tissues. This is the difference between pathogenic and non-pathogenic *Fusarium* strains, as the virulent strain causes irreversible changes in cell wall polymers during penetration of the epidermis.

In summary, the findings of this research strongly indicate that the reorganization of the cell wall emerges as one of the key factor contributing to flax sensitization induced by the non-pathogenic *Fusarium oxysporum* strain. Delving into the intricacies of these cellular mechanisms holds promise for the advancement of strategies aimed at fortifying flax’s resilience against fusarium wilt, ultimately leading to enhancements in both yield and quality. By comprehending and manipulating these underlying processes, there exists the potential to devise targeted interventions that not only mitigate the impact of *Fusarium oxysporum* but also pave the way for a more robust and productive cultivation of flax.

## Data availability statement

The raw data supporting the conclusions of this article will be made available by the authors, without undue reservation.

## Author contributions

WW: Conceptualization, Formal analysis, Funding acquisition, Investigation, Methodology, Project administration, Writing – original draft, Writing – review & editing. LD: Investigation, Methodology, Writing – review & editing. JH: Investigation, Methodology, Writing – review & editing. MB: Investigation, Writing – review & editing. AB: Investigation, Methodology, Writing – review & editing. JS: Conceptualization, Supervision, Writing – review & editing. AK: Conceptualization, Supervision, Writing – review & editing. JM: Formal Analysis, Investigation, Methodology, Writing – original draft, Writing – review & editing.
