# Supplementary material for: Endophytic non-pathogenic Fusarium oxysporum reorganizes the cell wall in flax seedlings
Source: Front Plant Sci. 2024 Mar 25;15:1352105. doi: 10.3389/fpls.2024.1352105 (PMC10999547; doi:10.3389/fpls.2024.1352105)
Supplement: Supplementary file 1 [file DataSheet_1.docx]

Supplementary Material

# Supplementary Figures and Tables

## Supplementary Figure 1

| **3 h** | **6 h** |
| --- | --- |
| 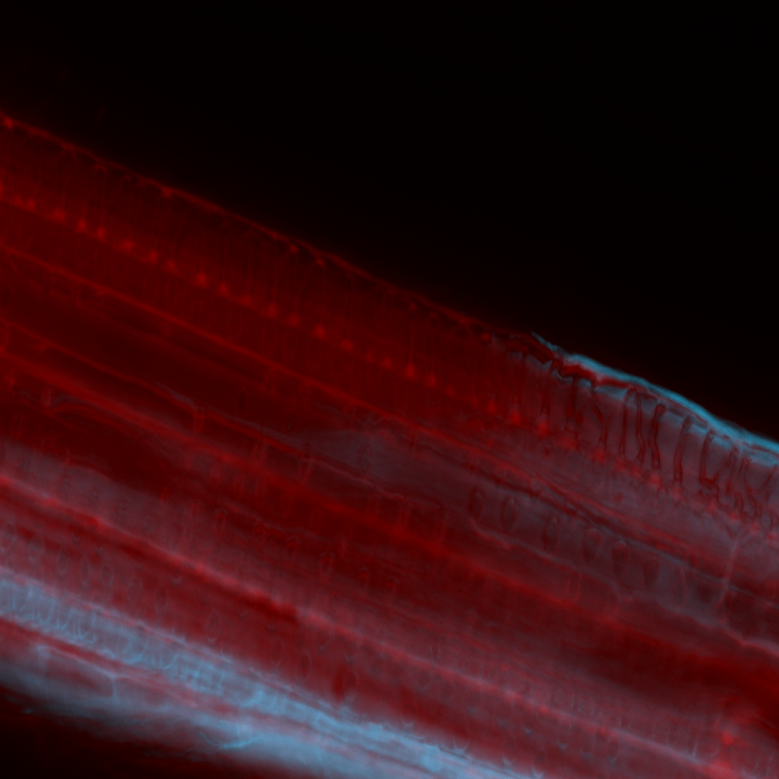 | 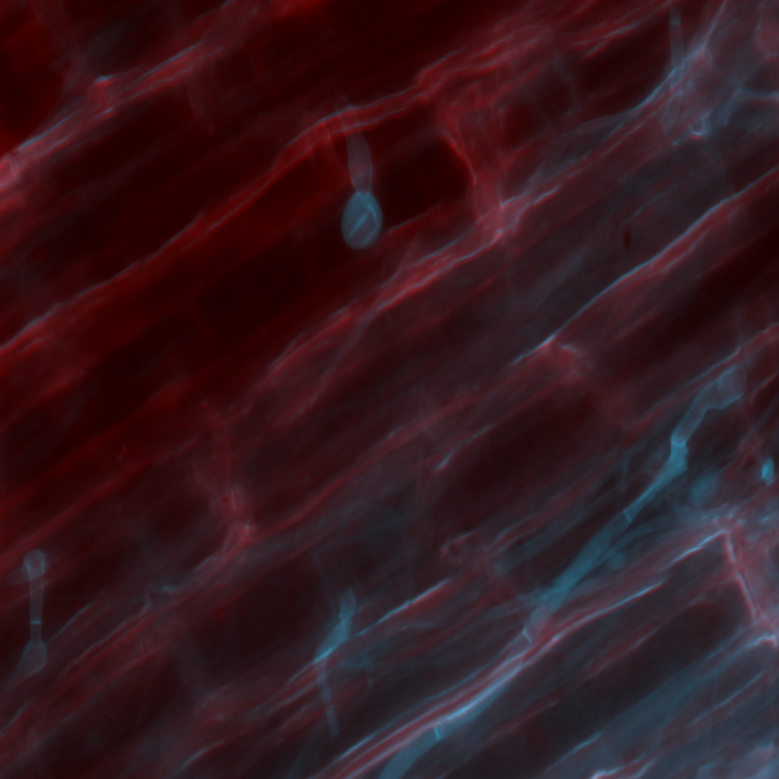 |
| **12 h** | **24 h** |
| 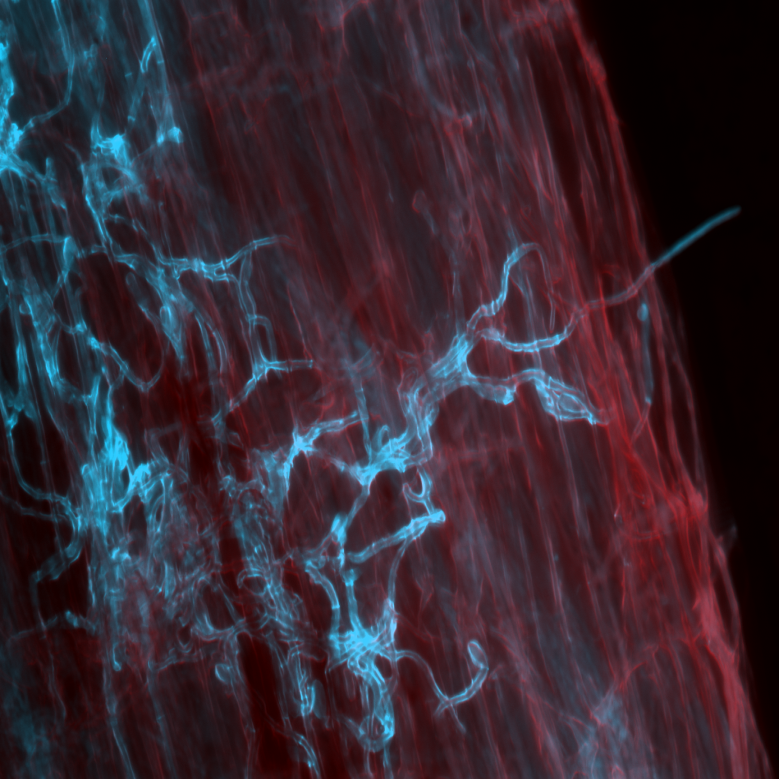 | 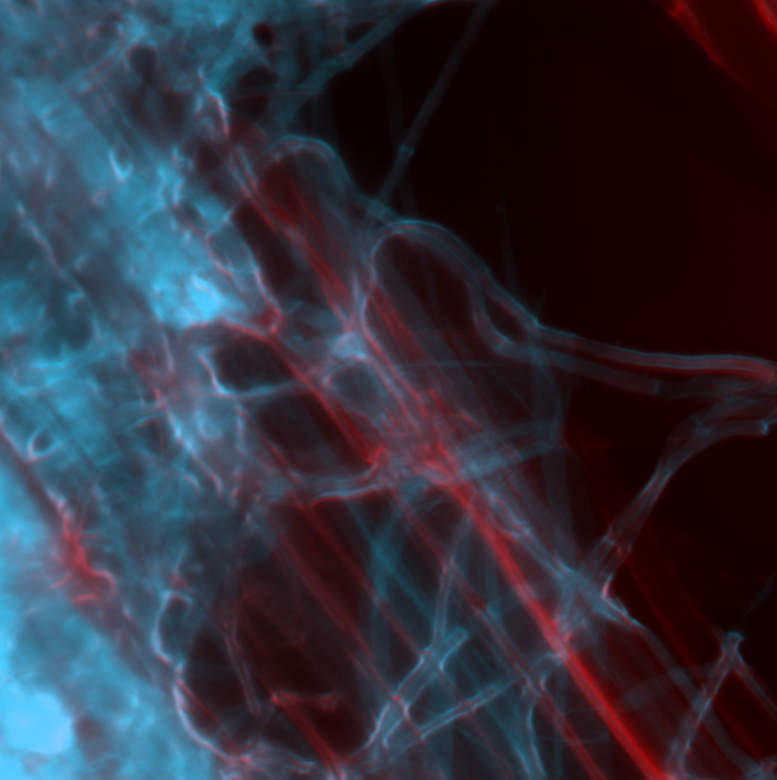 |
| **36 h** | **48 h** |
| 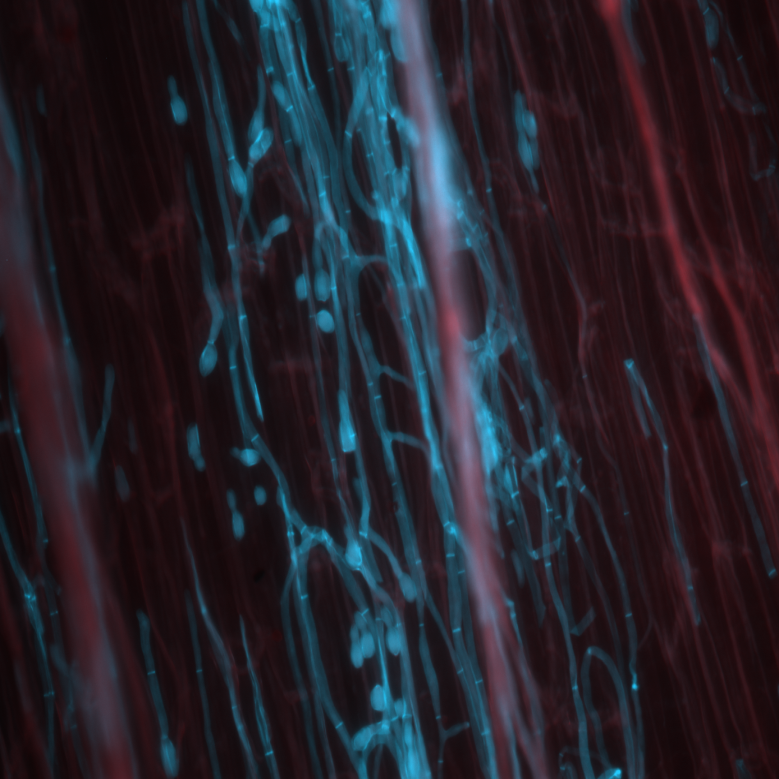 | 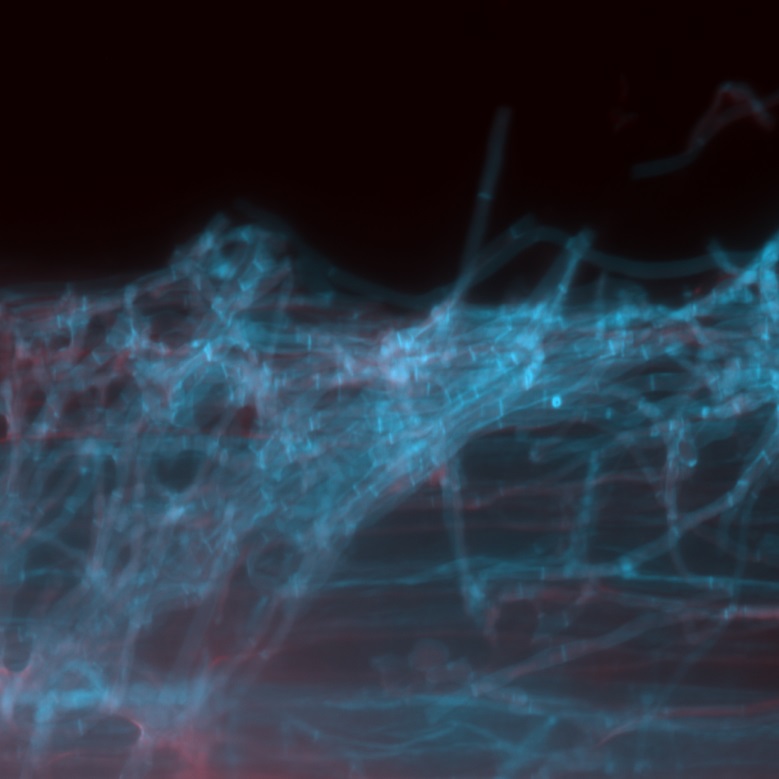 |

**Supplementary Figure 1.** Visualization of hyphae of the non-pathogenic strain *Fusarium oxysporum* attacking the roots of flax seedlings at 3, 6, 12, 24, 36 and 48 hours after treatment. Hyphae, stained with fluorescence dye - solophenyl flavine 7GFE (blue color on the image). The red color comes from safranin and mainly visualizes plant cell walls. Pictures were taken at 40 x magnification.

## Supplementary Figure 2

⃰

⃰

⃰

⃰

⃰

⃰

⃰

⃰

⃰

⃰

⃰

**Supplementary Figure 2.** The mRNA level of PR genes in flax seedlings in response to a non-pathogenic strain of *Fusarium oxysporum*. Changes in PR gene transcript levels (β-1,3 glucanases 1 and 2 and chitinase) in Nike flax seedlings treated with a non-pathogenic strain of *Fusarium oxysporum* over time (3 h, 6 h, 12 h, 24 h, 36 h and 48 h) are presented as relative amount (RQ) in relative to the reference gene (actin) for controls. Results were obtained by real-time PCR on a cDNA template and are presented as mean ± SD (n=3). The Student's t-test was used to determine the statistical significance of the obtained results (* -P < 0.05)

## Supplementary Figure 3


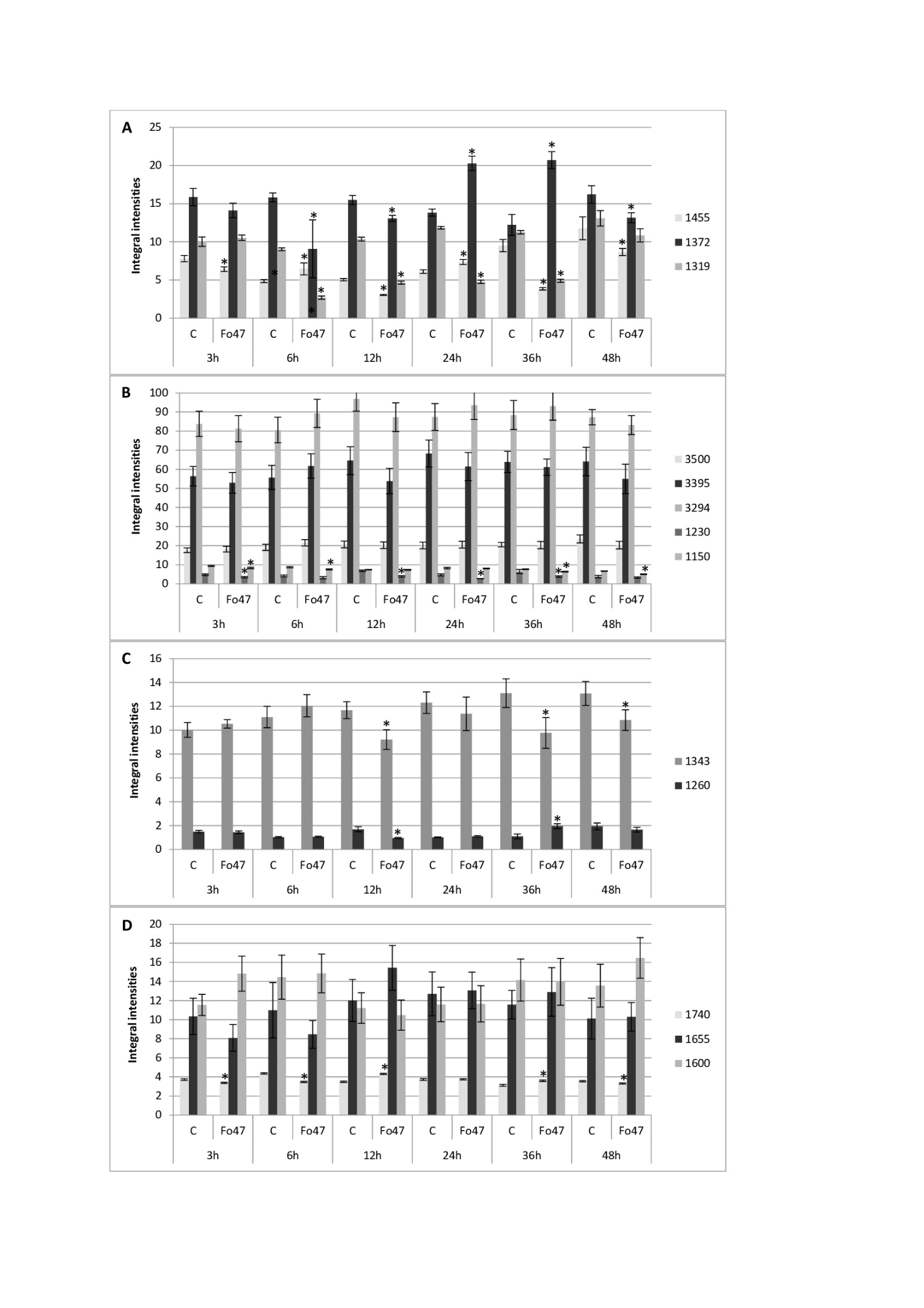

**Fig. S3.** Analysis of the integral intensities of FT-IR spectra bands of flax seedlings infected with non-pathogenic *Fusarium oxysporum* (Fo47). A). Changes in the integral intensities of bands at 1455 cm^-1^, 1372 cm^-1^ and 1319 cm^-1^. B). Changes in the integral intensities of bands at 3500 cm^-1^, 3395 cm^-1^, 3294 cm^-1^, 1230 cm^-1^ and 1150 cm^-1^. C). Changes in the integral intensities of bands at 1343 cm^-1^ and 1260 cm^-1^. D). Changes in the integral intensities of bands at 1740 cm^-1^, 1655 cm^-1^ and 1600 cm^-1^.

## Supplementary Figures 4

*

*

*

*

*

*

*

*

*

*

*

*

*

*

*

*

**Supplementary Figure 4.** The mRNA level of cellulose metabolism genes in flax seedlings in response to a non-pathogenic strain of *Fusarium oxysporum*. Changes in transcript levels of the cellulose synthesis (cellulose synthase isoforms 1-5) and cellulose degradation (cellulase 1 and cellulase 2) genes in Nike flax seedlings treated with a non-pathogenic strain of *Fusarium oxysporum* over time (3 h, 6 h, 12h, 24 h, 36 h and 48 h) are shown as relative quantity (RQ) to the reference gene (actin) for control. Results were obtained by real-time PCR on a cDNA template and are presented as mean ± SD (n=3). The Student's t-test was used to determine the statistical significance of the obtained results (* -P < 0.05)

## Supplementary Figure 5

*

*

*

*

*

*

*

*

*

*

*

*

*

*

*

*

*

*

*

*

*

*

*

*

*

*

*

*

*

*

*

**Supplementary Figure 5.** The mRNA level of hemicellulose metabolism genes in flax seedlings in response to a non-pathogenic strain of *Fusarium oxysporum*. Changes in gene transcript levels: hemicellulose synthesis (glucomannan 4-β-mannosyltransferase, galactosyltransferase galactomannan and xyloglucan xylosyltransferase) and hemicellulose degradation (endo-1,4-β-xylanase, 1,4-α-xylosidase, 1,4-β-xylosidase , α-galactosidase, endo-β-mannosidase and β-glycosidase) in Nike flaxseed seedlings treated with a non-pathogenic *Fusarium oxysporum* strain over time (3 h, 6 h, 12 h, 24 h, 36 h and 48 h) are presented as the relative amount (RQ) in relation to the gene (actin) for the control. Results were obtained by real-time PCR on a cDNA template and are presented as mean ± SD (n=3). The Student's t-test was used to determine the statistical significance of the obtained results (* -P < 0.05)

## Supplementary Figure 6

*

*

*

*

*

*

*

*

*

*

*

*

*

*

*

*

*

*

*

*

*

*

*

*

*

*

*

*

*

*

*

*

*

*

*

**Supplementary Figure 6.** The mRNA level of pectin metabolism genes in flax seedlings in response to a non-pathogenic strain of *Fusarium oxysporum*. Changes in gene transcript levels: pectin synthesis (UDP-D glucuronide epimerase 4, galacturonate transferase 1, galacturonate transferase 7, rhamnogalacturonate xylose transferase II, arabinose transferase and xylose transferase) and pectin degradation (pectin methylesterase 1, pectin methylesterase 3, pectin methylesterase that 5, polygalacturonase, pectin lyase I and pectin lyase II) in Nike flax seedlings treated with a non-pathogenic strain of *Fusarium oxysporum* over time (3 h, 6 h, 12 h, 24 h, 36 h and 48 h) are presented as the relative amount (RQ) in relation to the reference gene (actin) in regarding control. Results were obtained by real-time PCR on a cDNA template and are presented as mean ± SD (n=3). The Student's t-test was used to determine the statistical significance of the obtained results (* -P < 0.05).

## Supplementary Figure 7

*

*

*

*

*

*

*

*

*

*

*

*

*

*

*

*

*

*

*

*

*

*

*

*

*

*

*

*

*

*

*

*

*

*

**Supplementary Figure 7.** The mRNA level of lignin metabolism genes in flax seedlings in response to a non-pathogenic strain of *Fusarium oxysporum*. Changes in lignin metabolism gene transcript levels: ammonia-phenylalanine lyase, 4-hydroxycinnamoyl:CoA ligase, chalcone synthase, p-hydroxycinnamoylCoA:shikimic/quinonic acid hydroxycinnamoyltransferase, p-coumaric acid 3-hydroxylase, caffeoylCoA O-methyltransferase, 3/5-O -caffeic acid/5-hydroxyphenolic acid methyltranserase, sinapine alcohol dehydrogenase, hydroxycinnamic alcohol dehydrogenase and glucose transferase in Nike flax seedlings treated with a non-pathogenic *Fusarium oxysporum* strain over time (3 h, 6 h, 12 h, 24 h, 36 h and 48 h) are presented as relative amount (RQ ) relative to the reference gene (actin) for control. Results were obtained by real-time PCR on a cDNA template and are presented as mean ± SD (n=3). The Student's t-test was used to determine the statistical significance of the obtained results (* -P < 0.05)

## Supplementary Table 1

1. CELLULOSE METABOLISM

| **GENE** | **FORWARD PRIMER** | **REVERSE PRIMER** |
| --- | --- | --- |
| CSL1 | GGTCGAAATCTTCTTCTCACG | CGAGAATAAAGAGAGTGCGG |
| CSL2 | CTTACTCGCCAGTCCAAG | GTCACGTTGAGAGTTGACAC |
| CSL3 | AGTAGTAGGCTCAAGTTCCGA | GAATGCTATGTTTAGAGACTGGAC |
| CSL4 | TAATGTTGGCATCTACCCTTTC | CATTTGATCTCTAGGACGGC |
| CSL5 | TCAATGTCGGCATCTACCC | TGGCTAGACCAATGAGGC |
| CEL1 | CCATTACCCAAAGCACGTC | CTCGGTGTAGTTGTAGTTCATTC |
| CEL2 | AAGAGGCAAGTGGACTAC | GCCGATTTATAATAAACCGAGC |

1. HEMICELLULOSE METABOLISM

| **GENE** | **FORWARD PRIMER** | **REVERSE PRIMER** |
| --- | --- | --- |
| GMT | GCTAGAGTGCCAAAGGTG | GACCGATGTCCGAGTTATG |
| GGT | GTGTTTCTGATTCGTAACTGC | GAGTAAGTAGATCAACGCCG |
| XXT | AGATCGATTATTGCAGGCT | GCATCACTGTCCATCCAC |
| XYN | ACAGCAAGGAAGTCTTAACTAC | TCACAGCTTTCATTAAGTCATCT |
| XYL b | AAAGAACACCAGGATCTAGC | CTGCATAGTTTCCTAGAAGAGTC |
| XYL a | CAAGTATGATGATCAGCCTTTC | ATCTCGTTCATGTCGATCC |
| GS | CAGTCTCCTGGCGTGTTA | CAACTTTGACTCCATCTATGC |
| MS | TGACTATCTCGATGGCACAC | GGCATTCCTACAGAGCCAA |
| GLS | GATAAGGCCTCCGGATAAC | ATGAATACAGTGAATCGGGAC |

1. PECTIN METABOLISM

| **GENE** | **FORWARD PRIMER** | **REVERSE PRIMER** |
| --- | --- | --- |
| GAE | CCGAGGTACTCTTTCCTGA | CATACTTCTCGTTTACGAGGAAT |
| GAU 1 | AATTTAAGTGGCTTAATTCATCCTAC | GTGATTGAGCATTGAAAGATACTTG |
| GAU 7 | TGCAATATCCAAGGCACAGTTA | AGACAATGATGGCTCTTAGG |
| ARAD | ATTAACCAAGTATCCCGGC | TACTCCGTCAGCATGCAG |
| RGXT | TCCTGCGCTTGATTCACA | GGATCTCCCAGCCAAAC |
| PMT | ACGATTCTTGATATAGGCTGT | AGAACCAATCATCGCCG |
| PME1 | GAGCTGGAACCACATGC | AGCCTTCACGTATATTACGC |
| PME3 | AGTGGGATGGCAACTTT | TGAACCGACCGGGAGTA |
| PME5 | CGTAGTGGGTGACAGATTTAT | GAGGGAGTGGACGTAGAG |
| PG | GGATCAAGACTGCTGTGG | CAACAGGGATCGCGTTAG |
| PLL | GAGCATGTGATCGTATGCAA | CCTGGAAATGGTAATGTCAGT |
| PL | AACTACGAGAGAATCAAAGGAAC | GGGAGTATTCAACTCTCCG |

1. LIGNIN METABOLISM

| **GENE** | **FORWARD PRIMER** | **REVERSE PRIMER** |
| --- | --- | --- |
| PAL | GTTCTG TTTGAA GCCAAT GT | TGTAAG CACTCC CGTCG |
| 4CL | GCAGAAATGAAGATCGTCG | GTATGTAACCACCCTTGCT |
| CHS | AAATGG GGAGAA TGGAAG GA | CGCACG ATTCAA ATAGTG AGA |
| HCT | GTCGATATTCAAGCTGACCC | GTGGCGATGTACAGTTTAGT |
| C3H | CCATTGGAGTTCAAACCAGA | AGCAAGTGTCCCAACATAG |
| CCOACMT | CGGACAAGGACAACTACAT | ACGAAGTCCCTGTAGTACCTAA |
| COMT | CTCTTGGCTTCTTACTCTGTT | TGAGGACTTTGTCCTGGTT |
| SAD | CTACCTACGGAGGCTACT | GCTTGTCTAGCCCATAGAAC |
| GT | ATGCAGTGTGCATTCCAT | CGAATCGGAACGAAGGTAG |

1. PR GENES AND ACTIN

| **GENE** | **FORWARD PRIMER** | **REVERSE PRIMER** |
| --- | --- | --- |
| β-1,3-GLU1 | CTAGGCAGCGTGAAAGC | CGTCGAAGAGGTTGGTG |
| β-1,3-GLU2 | GATCTGGTCAAAGAGGTTGGTATAA | GTCCTTCTTCTTCCTCGATG |
| CHIT | CATCCAATGAATGGCCTT | GGCTGTTCGGAATGATATCTC |
| ACT | CCGGTGTTATGGTTGGAAT | TGTAGAAAGTGTGATGCCAAA |

4CL, 4-hydroxycinnamoyl-CoA ligase; ACT, actin; ARAD, arabinose transferase; CAD, cinnamic alcohol dehydrogenase; CCOACMT, caffeoyl-CoA O-methyltransferase, caffeic/5-hydroxyphenolic acid 3,5-O-methyltransferase; CEL, cellulase; CHIT, chitinase; CHS, chalcone synthase; COMT, Caffeoyl-CoA O-methyltransferase CSL, cellulose synthase; GAE, UDP-D-glucuronate 4-epimerase; EFF%, amplification efficiency %; GAU, galacturonate transferase; GGT, galactomannan galactosyltransferase; GLS, α-galactosidase GMT, glucomannan 4-β-mannosyltransferase; GS, β-glycosidase; GT, glucosyltransferase; HCT, p-hydroxycinnamoyl-CoA:shikimic/quinic acid transferase; MS, endo-β-mannosidase; PAL, phenylalanine ammonia lyase; PG, polygalacturonase; PL, pectin lyase; PLL, pectate lyase; PME, pectin methylesterase; PMT, pectin methyltransferase; RGXT, xylose:rhamnogalacturonate transferase II; SAD, sinapic alcohol dehydrogenase; XXT, xyloglucan xylosyltransferase; XYL a, 1,4-α-xylosidase; XYL b,1,4-β-xylosidase; XYN, endo-1,4-β-xylanase; β-1,3-GLU, β-1,3-glucanase

**Supplementary Table 1.** Primer sequences for real-time RT-PCR reactions. Primer sequences designed for real-time PCR: (A) Cellulose metabolism genes. (B) Hemicellulose metabolism genes (C) Pectin metabolism genes. (D) Lignin metabolism genes. (E) PR genes and actin gene.
